# An Epidemiological Human Disease Network Derived from Disease Co-occurrence in Taiwan

**DOI:** 10.1038/s41598-018-21779-y

**Published:** 2018-03-14

**Authors:** Yefei Jiang, Shuangge Ma, Ben-Chang Shia, Tian-Shyug Lee

**Affiliations:** 1 Graduate Institute of Business Administration, College of Management, Fu Jen Catholic University, New Taipei City, 24205 Taiwan; 2 Yale School of Public Health, New Haven, Connecticut United States of America; 3 College of Management, Taipei Medical University, Taipei, 11031 Taiwan

## Abstract

In “classic” biomedical research, diseases have usually been studied individually. The pioneering human disease network (HDN) studies jointly consider a large number of diseases, analyse their interconnections, and provide a more comprehensive description of diseases. However, most of the existing HDN studies are based on molecular information and can only partially describe disease interconnections. Building on the unique Taiwan National Health Insurance Research Database (NHIRD), in this study, we construct the epidemiological HDN (eHDN), where two diseases are concluded as interconnected if their observed probability of co-occurrence deviating that expected under independence. Advancing from the existing HDN, the eHDN can also accommodate non-molecular connections and have more important practical implications. Building on the network construction, we examine important network properties such as connectivity, module, hub, and others and describe their temporal patterns. This study is among the first to systematically construct the eHDN and can have important implications for human disease research and health care and management.

## Introduction

In “classic” biomedical research, diseases have usually been studied individually. Accumulating evidences have shown that diseases can be interconnected. For example, epidemiological studies have suggested the correlation between asthma and certain type of cancers^1^. Mutations in certain gene pathways, such as DNA repair and apoptosis, can lead to an elevated risk of multiple cancer types, making them “correlated”. In some early studies, a small number of pre-selected diseases were studied. A breakthrough is the pioneering human disease network (HDN) research^2–6^, under which a large number of diseases are simultaneously considered, and their interconnections are modelled.

Promising findings have been made in the HDN and other pan-disease studies. Notable studies include Calvano *et al*., which explored the genome-wide interaction network and suggested that network analysis using comprehensive knowledge can identify new functional modules perturbed in the disease processes^7^. Feldman *et al*. investigated the network properties of complex disease genes and found that network neighbours of known disease genes form an important class of candidates for identifying novel genes for the same disease^8^. Hidalgo *et al*. integrated different genetic, proteomic, and metabolic datasets, proposed a Phenotypic Disease Network, and found that disease progression can be represented and studied using network methods, offering the potential to enhance our understanding of the origin and evolution of human diseases^3^. Barabási *et al*. found that network medicine is essential for identifying new disease genes, for uncovering the biological significance of disease-associated mutations, and for identifying drug targets and biomarkers for complex diseases^9^.

Many of the recent HDN and other pan-disease studies, including the aforementioned, are based on molecular information. Such studies, despite significant successes, may have limitations. Most, if not all, diseases are only partially molecular. Shared environmental exposures, socioeconomic risk factors, and others can also lead to correlations among diseases. However, such non-molecular correlations cannot be effectively reflected in the existing HDNs. Shared molecular risk factors can only suggest the potential correlation in disease occurrence. That is, two diseases sharing common molecular risk factors not necessarily have a significantly higher (or lower) probability of co-occurrence, which may make the molecular HDNs practically less relevant. In addition, searching for the molecular basis is still an ongoing effort for most diseases, which may cast concerns on the credibility of the molecular HDNs.

There are also a few studies that establish disease interconnections based on clinical and epidemiological data. However, with constrained data availability, they are often limited to a small number of diseases and possibly biassed sample selection^10^.

The goal of this study is to construct the epidemiological HDN (eHDN), where two diseases are concluded as connected if their probability of co-occurring in clinics deviating from that expected under independence. This effort will take advantage of the unique Taiwan National Health Insurance Research Database (NHIRD; more details below). The eHDN fits the HDN analysis paradigm and will have similar important implications as the existing HDNs. On the other hand, it may advance from the existing literature in multiple aspects. Built on data observed in clinics, it can accommodate both molecular and non-molecular disease connections and hence be more comprehensive. By directly built on observed disease occurrence, it can be practically more relevant. In addition, with the huge sample size of NHIRD, the constructed network can be more reliable than some of the existing ones. Overall, this eHDN analysis may complement the existing molecular HDNs and significantly advance our understanding of disease interconnections from an epidemiological perspective. It may provide important insights for health care and management.

## Methods

### Database

Taiwan launched the single-payer national health insurance (NHI) programme on March 1st, 1995. By the end of 2004, about 99.9% of the Taiwan population were enrolled^11,12^. With the high cost of treatments that are not insured or by commercial insurance, the dominating majority of hospital/clinic-based disease treatments go through NHI. To get insurance reimbursement, hospitals and clinics are required to provide comprehensive data on each disease treatment episode. Data are then sorted and stored in NHIRD. Compared to other databases, unique advantages of the NHIRD may include unbiasedness (virtually the whole Taiwan population are covered), comprehensiveness (comprehensive information are available on all inpatient and outpatient treatment episodes), and uniformity (all data are collected and stored under the same protocol). NHIRD has served as the basis of a large number of biomedical and public health studies (with already close to 400 publications in PubMed). We refer to Hwang *et al*. and the NHIRD website for more detailed information on NHIRD^11,13^.

In this study, we retrieved data collected between 2000 and 2013 from NHIRD. The initial dataset contains records on one million subjects (about 4.26% of Taiwan’s population) randomly selected from the 2005 registry for beneficiaries. In NHIRD, each subject has a unique ID, which is used to link different databases. For our analysis, we analysed both outpatient and inpatient treatments, with information in the CD (ambulatory care expenditures by visits) and DD (inpatient expenditures by admissions) files, respectively.

For disease identification, the ICD-9-CM code was used. Prior to 2005, the ICD-9-CM 1992 version was used. For consistency, it was converted to the 2001 version. With more interest on diseases, following the literature, records with the E and V codes (external causes of injury and supplemental classification), 630–679 (Pregnancy, Childbirth and Puerperium Complications), and 760–999 (Symptoms, Signs & Ill-Defined Conditions) of ICD-9-CM were removed from analysis. Limitations of the ICD-9 code have been recognised. For example, it may be biassed by experts’ discrimination. In addition, the vocabulary used to describe multiple patient billing codes may actually describe the same clinical disease. To address such problems, following the literature^14^, we adopted the electronic health record (EHR) driven Phenome-wide association studies (PheWAS) codes (PheCode), which group the ICD-9 codes into 1,723 PheWAS Codes (PheCode). To generate more reliable estimates, we focused on common diseases defined as having nonzero occurrence in each of the fourteen calendar years, leading to a total of 1,356 diseases for downstream analysis. More information on data processing is provided in Supplementary Information (SI). On the patient side, records with inconsistency (for example, conflicting sex information) were removed to ensure a high standard of analysis. The final analysed dataset contains records on 986,646 patients with 1,381,749 inpatient and 173,355,725 outpatient episodes in the study period. Among them, there are 486,992 males and 499,654 females. More information is provided in SI.

This study was exempt from full review by the Institutional Review Board of Fu Jen Catholic University, as only de-identified data are analysed for a research purpose.

### Network-based Analysis

**Figure 1:**
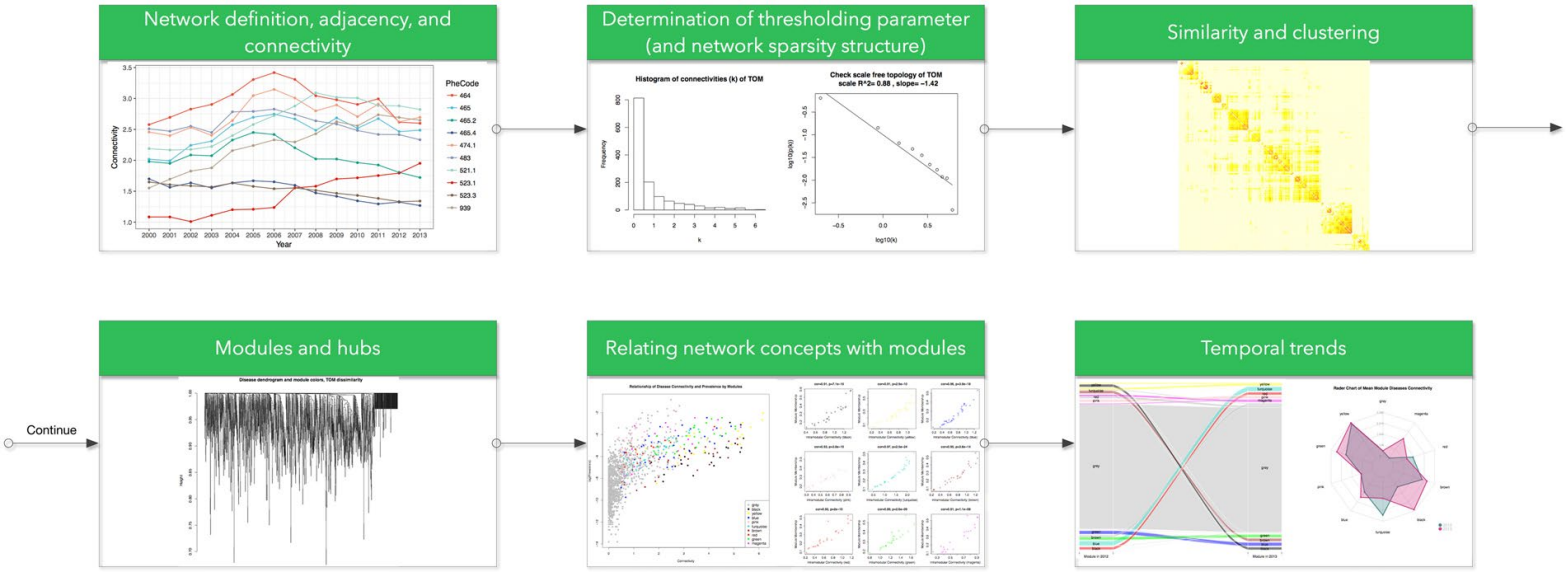
Flowchart of network-based analysis.

Our network analysis is based on the WGCNA (weighted gene co-expression network analysis)^15^, which was originally designed for the analysis of gene expression data and has demonstrated satisfactory performance in a large number of publications^16–19^. A closer examination of WGCNA suggests that its applicability is not limited to gene expression data. For the completeness of this article, below the analysis steps are briefly described, and readers are referred to Horvath for more details^20^. It is noted that although with some minor changes, the main advancement of this study is not on the WGCNA technique itself. Rather, this study marks a new and innovative application of the WGCNA technique. A flowchart describing the proposed analysis procedure is shown in Fig. 1. Below we provide more details on each analysis step.

#### Network Construction

In the eHDN analysis, a node corresponds to a disease, and two diseases are connected with an edge if their probability of co-occurrence deviating from that expected under independence. The edge information is accommodated in the adjacency matrix. Denote *n* as the number of diseases. For diseases *i* and *j* (*i*, *j* ∈ 1, …, *n*), their *ϕ*-correlation is computed as:

1 $${s}_{ij}=\frac{{C}_{ij}N-{P}_{i}{P}_{j}}{\sqrt{{P}_{i}{P}_{j}(N-{P}_{i})\,(N-{P}_{j})}}$$

where, for a fixed time period, *C*_*ij*_ is the number of patients with both diseases (treated in the same or different, inpatient or outpatient, episodes), *N* is the total number of patients, and *P*_*i*_ and *P*_*j*_ are the number of patients with diseases *i* and *j*. Denote *S* = [*s*_*ij*_] as the *n* × *n* similarity matrix with its (*i*, *j*)th element being *s*_*ij*_. Then the *n* × *n* adjacency matrix *A* can be defined where (*i*, *j*)th element is

2 $${a}_{ij}=Ad\,jFunc({s}_{ij},\tau )\equiv \{\begin{array}{ll}|{s}_{ij}| & if\,{s}_{ij}\ge \tau \\ 0 & if\,{s}_{ij} < \tau \end{array}$$

Here the threshold *τ* is imposed to remove spurious small correlations, only retain the large ones, and generate a sparse and more interpretable network. Its value is chosen using the scale-free topology criterion^2,3,15,21^, which has been extensively adopted in the literature. In the adjacency matrix, all components take values between 0 and 1 (that is, positive and negative correlations are treated in the same manner). Two diseases are more strongly correlated (positive or negative) if their corresponding value in the adjacency matrix is bigger.

#### Connectivity, Module, and Hub

For node (disease) *i*, its connectivity is defined as ${K}_{i}={\sum }_{j\ne i}\,{a}_{ij}$, which quantifies how strongly it is connected to the other nodes. In the literature, an alternative definition of connectivity has also been considered, where ${k}_{i}={\sum }_{j\ne i}\,TO{M}_{ij}$ (the definition of TOM is provided below. more information on the two connectivity measures is provided in SI).

An important network concept is module (also referred to as “community” in some studies), which is composed of tightly connected nodes. Consider the topological overlap matrix (TOM), where its (*i*, *j*)th element is:

3 $$TO{M}_{ij}=\frac{{l}_{ij}+{a}_{ij}}{{\rm{\min }}\,\{{K}_{i}{K}_{j}\}+1-{a}_{ij}}$$

with ${l}_{ij}={\sum }_{u}\,{a}_{iu}{a}_{uj}$. Loosely speaking, *l*_*ij*_ measures how many neighbour nodes that *i* and *j* shared. *TOM*_*ij*_ measures the distance between diseases *i* and *j* in a network sense^20,22^. Accordingly, define *dissTOM*_*ij*_ = 1 − *TOM*_*ij*_, which is non-negative and symmetric and measures the dissimilarity between any two diseases. With matrix *dissTOM*, whose (*i*, *j*)th element is *dissTOM*_*ij*_, modules can be identified by hierarchical clustering with a dynamic tree cutting approach^15,23^.

With each module, connectivity can be re-computed and referred to as intramodular connectivity. Nodes (diseases) with the highest correlation with the eigen-diseases (definition below) are identified as hubs.

#### Remarks

The network quantities described above have important implications. Adjacency directly describes how strongly two diseases are connected to each other. Of interest are diseases that are tightly interconnected. In health care management and planning, such diseases should be considered together as opposed to individually. In network analysis, it has been suggested that more highly connected nodes play more important roles in a network. It is thus of interest to examine connectivity and identify the highly connected ones. Such nodes (diseases) may have a higher priority in disease control and prevention, as they can potentially have a higher impact on the overall health conditions. In biomedical research, clustering/classifying diseases is an important task, and the module structure provides an alternative way for disease clustering. Diseases within the same modules can potentially share common risk factors (and thus the analysis can have scientific value) and be treated with similar regimens (and thus the analysis can have practical value).

#### Temporal Trends

For all diseases, occurrence rates change over time. In addition, occurrence observed in clinics is also affected by diagnosis and other factors, which are also time-dependent. As such the eHDN and its quantities described above change over time. By conducting analysis year by year and comparing across time, we are able to obtain the temporal trends of the eHDN. This is significantly different from the molecular HDNs, which are static. For scalar quantities, variation over time can be directly assessed. For the module structure, we assess variation using the Jaccard indexes for modules obtained in consecutive years.

#### Visualisation

The patient-disease relationship and disease correlations can be visualised using heatmaps. The overall network structure can be visualised using the software Gephi^24^. In such a plot, diseases that share edges are connected with lines, the size of a node is proportional to its connectivity, and different modules are represented using different colours. Construction of modules can be visualised using dendrograms and heatmaps. For scalar quantities (prevalence, connectivity, etc.), changes over time can be visualised using scatter plots (possibly with nonparametric fits). Changes of module memberships can be visualised using alluvial diagrams. Changes of modulesâ€™ mean connectivity can be visualised using radar charts. The aforementioned visualisation tools provide a more intuitive way of interpreting network structures and properties.

## Results

The numbers of patients for each calendar year are shown in Table S1 in SI. In Table S2 in SI, we further present summary statistics on the numbers (proportions) of inpatient and outpatient treatment episodes, stratified by gender, age, and calendar year. Across time, an increasing trend of inpatient treatment is observed. For outpatient treatment, an increasing trend is also observed, although not as prominent as for inpatient. It is noted that the number of observations per year is large enough (larger than many of the peer studies) to make credible inference.

**Figure 2:**
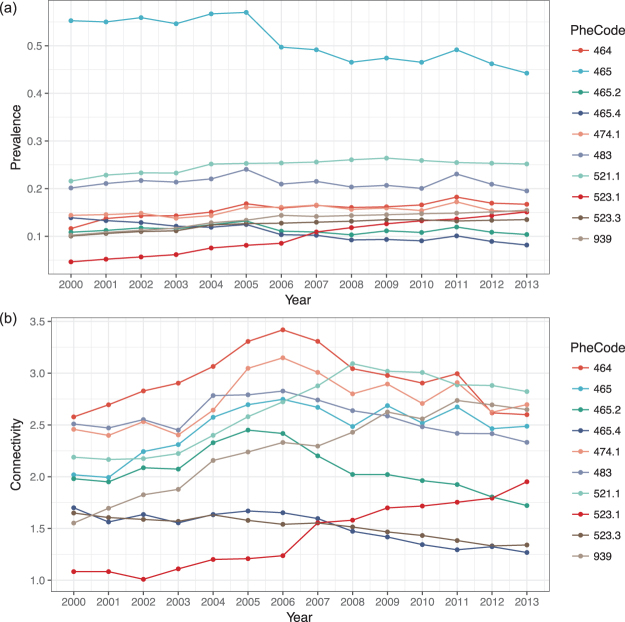
Top ten diseases with the highest prevalence (top) and their connectivity (bottom).

For a more intuitive description of the patient-disease distributions, in Fig. S2 in SI, we present the patient-disease heatmaps, where the x-axis corresponds to patients, the y-axis corresponds to diseases, and a red dot represents one disease occurrence. It is noted that with the huge sample size, plotting all patients generates plots with huge sizes. Thus, in Fig. S2, we presented results for 1% subjects randomly selected from our data in 2012 and 2013. The prevalences of diseases are computed year by year. The top ten are presented in the top panel of Fig. 2. Acute upper respiratory infections (code 465) has the highest prevalence in all years. The high prevalence of acute upper respiratory infections in Taiwan has been noted in multiple publications^25^. Also in the top ten are gingivitis (code 523.1), acute sinusitis (code 464), and atopic/contact dermatitis (code 939) and others, all of which have been extensively examined in the literature^13,25–27^.

### Network construction and connectivity

**Figure 3:**
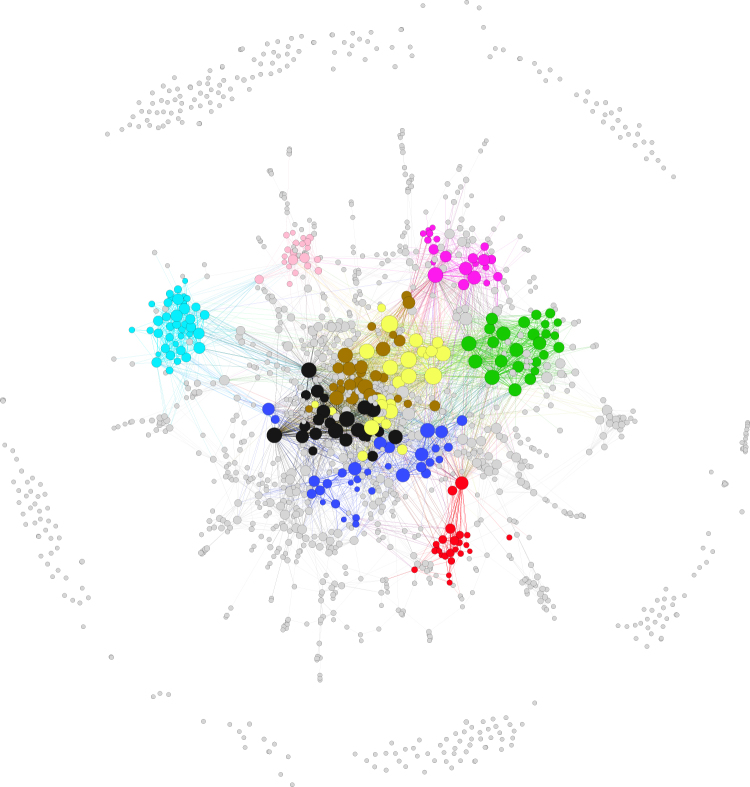
The traditional view of eHDN in the year 2013.

The eHDN is constructed using the approach described above. The threshold value *τ* is determined as 0.03. More detailed results are provided in SI. In Fig. 3, we provide a “traditional view” of the network structure of the eHDN for the year 2013. Similar constructions/plots have also been done for other years (details omitted and available from the authors). In Fig. 3, diseases that are connected with edges are linked with lines. Extensive “activities” are observed, suggesting a high degree of interconnections among diseases. A certain number of isolated diseases not linking to other diseases are also observed.

As shown in Fig. S4 in SI, for the NHIRD data, the weighted connectivity *k*_*i*_ and unweighted connectivity *K*_*i*_ values are highly correlated. In the bottom panel of Fig. 2, we present the weighted connectivity values for the top ten diseases. More variations are seen in connectivity than in prevalence. For multiple diseases, bell-shaped curves are observed. Such an observation has not been made in the literature. Changing in connectivity can be caused by both intrinsic reasons (such as changing patterns in disease occurrence) as well as reasons such as diagnosis. Disease 464 (acute sinusitis) is observed to have the highest connectivity. It is a common disease and related to a large number of respiratory diseases. Significant increases in connectivity are observed for multiple diseases, especially disease 523.1 (gingivitis) and disease 939 (atopic/contact dermatitis). As a common non-destructive gum disease, gingivitis has been increasingly linked to multiple oral, digestive, and blood diseases. Also, patients with atopic/contact dermatitis and allergic rhinitis have a higher risk of asthma and many autoimmune diseases^13,28^. More information on connectivity is also available in Fig. 3, where the sizes of nodes are proportional to their connectivity. The variations in connectivity across nodes are clearly observed.

In the investigation of disease connectivity, we first identify those with the highest overall connectivity across 2000 and 2013 and present the top ten diseases in the upper panel of Table 1. The list includes multiple heart diseases, type 2 diabetes, and osteoarthrosis, all of which have been suggested as connected to a large number of diseases. It is “reassuring” that our analysis coincides with “traditional wisdom”. In the lower panel of Table 1, we present the list of hub diseases for the year 2013. Their module information is also provided in the bracket. The differences between the upper and lower panels are caused by the module structure (hubs are identified within modules separately) as well as variations across years.

**Table 1:** Diseases with the highest overall connectivity (upper) and hubs in the year 2013 (lower).

Type of Disease	PheCode	Disease (module)	Connectivity	Intramodular Connectivity
Overall Top Ten Disease	401.1	Essential hypertension	6.11	1.63
	250.2	Type 2 diabetes	5.97	1.77
	401.21	Hypertensive heart disease	5.92	1.74
	411.8	Other chronic ischemic heart disease, unspecified	5.62	1.79
	411.4	Coronary atherosclerosis	5.37	1.83
	366.2	Senile cataract	5.32	0.82
	272.1	Hyperlipidemia	5.21	1.48
	740.9	Osteoarthrosis NOS	5.16	1.59
	433.8	Late effects of cerebrovascular disease	5.05	1.72
	600	Hyperplasia of prostate	4.98	1.24
Hub Disease in 2013	401.1	Essential hypertension (yellow)	6.16	1.04
	480	Pneumonia (black)	5.38	1.35
	366.2	Senile cataract (magenta)	5.27	0.76
	585.3	Chronic renal failure (brown)	5.14	1.20
	721.1	Spondylosis without myelopathy (green)	5.06	1.50
	626.13	Irregular menstrual cycle (turquoise)	3.59	2.30
	571.51	Cirrhosis of liver without mention of alcohol (blue)	3.21	1.28
	300.4	Dysthymic disorder (red)	2.70	1.13
	939	Atopic/contact dermatitis (pink)	2.65	0.93

### Module identification and properties

**Figure 4:**
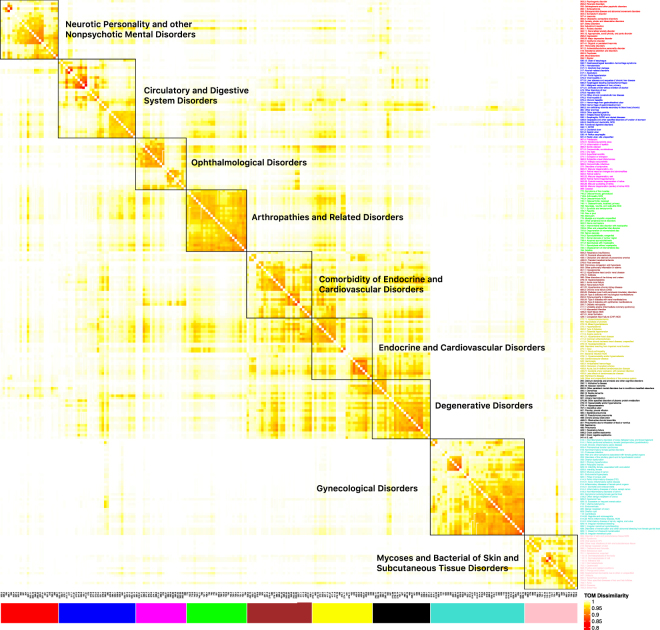
Disease module structure for the year 2013. Different modules are represented using different colours.

Construction of the module structure first involves constructing the dendrogram. In the left panel of Fig. S6 in SI, we show the dendrogram for the year 2013 as well as the identified modules. Different colours represent different modules, and the grey colour represents diseases not classified in the identified modules. Different modules are also represented using different colours in Fig. 3. In Fig. 4, we show the heatmap of the diseases and mark different modules using black boundaries. The “clustering structure” along the diagonal is clearly seen, which suggests the distinct differences across modules. For the year 2012, we show the corresponding plots in Fig. S9 in SI. A careful comparison of the plots suggests variation across time (more definitive results below). For the other years, similar plots can be generated (omitted here, available from the authors).

Different modules differ in multiple aspects. First, they have different sizes. The nine modules have sizes 22 (pink), 39 (turquoise), 21 (magenta), 24 (red), 32 (blue), 27 (brown), 25 (yellow), 24 (black), and 25 (green), respectively. Also, as can be seen from Fig. 4, the levels of connections within modules also vary. For example, there are tighter connections within the green module than others. Diseases in different modules also have different levels of connectivity. More detailed statistics on connectivity are provided in Fig. S5 in SI. From a biomedical perspective, it is of interest to examine the “meanings” of the modules. In Table 2, we provide the detailed list of diseases in the nine modules. As suggested in the published HDN studies, the modules provide an alternative way of defining disease classifications. More specifically, our classification, as shown in Table 2, is based on whether diseases co-occur on the same patients. An enrichment analysis is conducted to examine the representative diseases of different modules. It is found that the nine modules are enriched with the following diseases: mycoses and bacterial of skin and subcutaneous tissue disorders (pink); gynaecological disorders (turquoise); ophthalmological disorders (magenta); neurotic personality and other nonpsychotic mental disorders (red); circulatory and digestive system disorders (blue); comorbidity of endocrine and cardiovascular disorders (brown); endocrine and cardiovascular disorders (yellow); degenerative disorders (black) and arthropathies and related disorders (green), respectively. It is noted that some disease clustering/classification structures in the literature are based on, for example, biology and are defined for the whole population. In contrast, our network and module structure, based on the NHIRD, are tailored to the Taiwan population. The Taiwan population are dominatingly Asian, which may have disease risk and characteristics different from other populations. In addition, disease occurrence highly depends on environmental, socioeconomic, and other factors, which vary significantly across regions/countries. As such, for the Taiwan population and their health care and management, our constructed module structure/disease classification can be more sensible.

**Table 2:** Disease module structure for the year 2013.

Color	PheCode	Disease	Color	PheCode	Disease	Color	PheCode	Disease	Color	PheCode	Disease	Color	PheCode	Disease
pink	078	Viral warts & HPV	turquoise	624	Symptoms involving female genital tract	red	301	Personality disorders	brown	251.1	Hypoglycemia	black	038.2	Gram positive septicemia
pink	110.1	Dermatophytosis	turquoise	625	Pain and other symptoms associated with female genital organs	red	301.2	Antisocial/borderline personality disorder	brown	276.13	Hyperpotassemia	black	041.4	E. coli
pink	110.11	Dermatophytosis of nail	turquoise	626	Disorders of menstruation and other abnormal bleeding from female genital tract	red	303.3	Psychogenic disorder	brown	276.41	Acidosis	black	270.38	Other specified disorders of plasma protein metabolism
pink	110.12	Althete's foot	turquoise	626.1	Irregular menstrual cycle/bleeding	red	303.4	Somatoform disorder	brown	276.6	Fluid overload	black	276.12	Hyposmolality and/or hyponatremia
pink	110.13	Dermatophytosis of the body	turquoise	626.11	Absent or infrequent menstruation	red	304	Adjustment reaction	brown	401.2	Hypertensive heart and/or renal disease	black	276.14	Hypopotassemia
pink	216	Benign neoplasm of skin	turquoise	626.12	Excessive or frequent menstruation	red	316	Substance addiction and disorders	brown	401.22	Hypertensive chronic kidney disease	black	290	Delirium dementia and amnestic and other cognitive disorders
pink	686	Other local infections of skin and subcutaneous tissue	turquoise	626.13	Irregular menstrual cycle	red	327	Sleep disorders	brown	411.1	Unstable angina (intermediate coronary syndrome)	black	290.1	Dementias
pink	686.1	Carbuncle and furuncle	turquoise	626.14	Irregular menstrual bleeding	red	327.4	Insomnia	brown	411.2	Myocardial infarction	black	290.11	Alzheimer's disease
pink	686.5	Pyoderma	turquoise	626.15	Infertility, female, associated with anovulation	red	327.41	Organic or persistent insomnia	brown	427.21	Atrial fibrillation	black	290.13	Senile dementia
pink	689	Disorder of skin and subcutaneous tissue NOS	turquoise	626.2	Dysmenorrhea	red	333	Extrapyramidal disease and abnormal movement disorders	brown	428.1	Congestive heart failure (CHF) NOS	black	290.16	Vascular dementia
pink	690.1	Seborrheic dermatitis	turquoise	626.4	Premenstrual tension syndromes	blue	070.2	Viral hepatitis B	brown	428.2	Heart failure NOS	black	290.3	Other persistent mental disorders due to conditions classified elsewhere
pink	695.3	Rosacea	turquoise	626.8	Infertility, female	blue	070.3	Viral hepatitis C	brown	433.1	Occlusion and stenosis of precerebral arteries	black	480	Pneumonia
pink	695.7	Prurigo and Lichen	turquoise	628	Ovarian cyst	blue	070.4	Chronic hepatitis	brown	433.12	Cerebral atherosclerosis	black	480.1	Bacterial pneumonia
pink	698	Pruritus and related conditions	magenta	362.21	Macular degeneration, dry	blue	070.9	Hepatitis NOS	brown	433.31	Transient cerebral ischemia	black	480.12	Pseudomonal pneumonia
pink	701.1	Keratoderma, acquired	magenta	362.22	Macular degeneration, wet	blue	155.1	Malignant neoplasm of liver, primary	brown	503	Pulmonary congestion and hypostasis	black	496	Chronic airway obstruction
pink	704.8	Other specified diseases of hair and hair follicles	magenta	362.23	Cystoid macular degeneration of retina	blue	280.2	Iron deficiency anemia secondary to blood loss (chronic)	brown	505	Other pulmonary inflamation or edema	black	496.21	Obstructive chronic bronchitis
pink	705.1	Dyshidrosis	magenta	362.26	Macular puckering of retina	blue	285	Other anemias	brown	509.2	Respiratory insufficiency	black	501	Pneumonitis due to inhalation of food or vomitus
pink	706.1	Acne	magenta	362.29	Macular degeneration (senile) of retina NOS	blue	317	Alcohol-related disorders	brown	585.1	Acute renal failure	black	507	Pleurisy; pleural effusion
pink	706.2	Sebaceous cyst	magenta	362.4	Retinal vascular changes and abnomalities	blue	317.1	Alcoholism	brown	585.2	Renal failure NOS	black	509.1	Respiratory failure
pink	706.3	Seborrhea	magenta	362.8	Retinal hemorrhage/ischemia	blue	317.11	Alcoholic liver damage	brown	585.3	Chronic renal failure [CKD]	black	563	Constipation
pink	939	Atopic/contact dermatitis due to other or unspecified	magenta	362.9	Retinal edema	blue	530.1	Esophagitis, GERD and related diseases	brown	586	Other disorders of the kidney and ureters	black	591	Urinary tract infection
pink	947	Urticaria	magenta	366	Cataract	blue	530.11	GERD	yellow	041	Bacterial infection NOS	black	707.1	Decubitus ulcer
turquoise	112	Candidiasis	magenta	366.2	Senile cataract	blue	530.12	Ulcer of esophagus	yellow	250.2	Type 2 diabetes	green	351	Other peripheral nerve disorders
turquoise	131	Protozoan infection	magenta	367.4	Presbyopia	blue	530.14	Reflux esophagitis	yellow	272.1	Hyperlipidemia	green	353.2	Nerve root lesions
turquoise	218.1	Uterine leiomyoma	magenta	368.9	Subjective visual disturbances	blue	530.2	Esophageal bleeding (varices/hemorrhage)	yellow	272.11	Hypercholesterolemia	green	716.9	Arthropathy NOS
turquoise	218.2	Other benign neoplasm of uterus	magenta	369.5	Conjunctivitis, infectious	blue	530.7	Gastroesophageal laceration-hemorrhage syndrome	yellow	272.12	Hyperglyceridemia	green	720	Spinal stenosis
turquoise	220	Benign neoplasm of ovary	magenta	370.2	Superficial keratitis	blue	531.1	Hemorrhage from gastrointestinal ulcer	yellow	272.13	Mixed hyperlipidemia	green	720.1	Spinal stenosis of lumbar region
turquoise	253	Disorders of the pituitary gland and its hypothalamic control	magenta	370.31	Keratoconjunctivitis sicca	blue	531.2	Gastric ulcer	yellow	274.1	Gout	green	721.1	Spondylosis without myelopathy
turquoise	253.1	Pituitary hyperfunction	magenta	371.2	Conjunctivitis, noninfectious	blue	531.3	Duodenal ulcer	yellow	274.11	Gouty arthropathy	green	721.2	Spondylosis with myelopathy
turquoise	256	Ovarian dysfunction	magenta	371.21	Allergic conjunctivitis	blue	531.4	Peptic ulcer, site unspecified	yellow	276.11	Hyperosmolality and/or hypernatremia	green	722.1	Displacement of intervertebral disc
turquoise	256.4	Polycystic ovaries	magenta	371.3	Inflammation of eyelids	blue	535.8	Other specified gastritis	yellow	332	Parkinson's disease	green	722.6	Degeneration of intervertebral disc
turquoise	614	Inflammatory diseases of female pelvic organs	magenta	372	Disorders of conjunctiva	blue	535.9	Gastritis and duodenitis, NOS	yellow	342	Hemiplegia	green	722.7	Intervertebral disc disorder with myelopathy
turquoise	614.1	Pelvic peritoneal adhesions, female (postoperative) (postinfection)	magenta	374.1	Ectropion or entropion	blue	536.8	Dyspepsia and other specified disorders of function of stomach	yellow	349	Other and unspecified disorders of the nervous system	green	722.9	Other and unspecified disc disorder
turquoise	614.3	Pelvic inflammatory disease (PID)	magenta	375.1	Dry eyes	blue	564	Functional digestive disorders	yellow	401.1	Essential hypertension	green	727.1	Synovitis and tenosynovitis
turquoise	614.31	Acute inflammatory pelvic disease	red	295	Schizophrenia and other psychotic disorders	blue	564.1	Irritable Bowel Syndrome	yellow	401.21	Hypertensive heart disease	green	728.7	Fasciitis
turquoise	614.32	Chronic inflammatory pelvic disease	red	295.1	Schizophrenia	blue	571.5	Other chronic nonalcoholic liver disease	yellow	411.3	Angina pectoris	green	738.4	Acquired spondylolisthesis
turquoise	614.33	Pelvic inflammatory disease, NOS	red	295.2	Paranoid disorders	blue	571.51	Cirrhosis of liver without mention of alcohol	yellow	411.4	Coronary atherosclerosis	green	740.1	Osteoarthritis; localized
turquoise	614.4	Inflammatory diseases of uterus, except cervix	red	295.3	Psychosis	blue	571.8	Liver abscess and sequelae of chronic liver disease	yellow	411.8	Other chronic ischemic heart disease, unspecified	green	740.11	Osteoarthrosis, localized, primary
turquoise	614.5	Inflammatory disease of cervix, vagina, and vulva	red	296	Mood disorders	blue	571.81	Portal hypertension	yellow	430.2	Intracerebral hemorrhage	green	740.2	Osteoarthrosis, generalized
turquoise	614.51	Cervicitis and endocervicitis	red	296.1	Bipolar	blue	573	Other disorders of liver	yellow	433	Cerebrovascular disease	green	740.9	Osteoarthrosis NOS
turquoise	614.52	Vaginitis and vulvovaginitis	red	296.2	Depression	blue	578.1	Hematemesis	yellow	433.2	Occlusion of cerebral arteries	green	745	Pain in joint
turquoise	615	Endometriosis	red	296.22	Major depressive disorder	blue	578.9	Hemorrhage of gastrointestinal tract	yellow	433.21	Cerebral artery occlusion, with cerebral infarction	green	754.2	Spondylolisthesis, congenital
turquoise	619	Noninflammatory female genital disorders	red	300	Anxiety, phobic and dissociative disorders	brown	250.22	Type 2 diabetes with renal manifestations	yellow	433.6	Acute, but ill-defined cerebrovascular disease	green	760	Back pain
turquoise	619.1	Noninflammatory disorders of ovary, fallopian tube, and broad ligament	red	300.1	Anxiety disorder	brown	250.23	Type 2 diabetes with ophthalmic manifestations	yellow	433.8	Late effects of cerebrovascular disease	green	764	Sciatica
turquoise	619.3	Noninflammatory disorders of cervix	red	300.11	Generalized anxiety disorder	brown	250.24	Type 2 diabetes with neurological manifestations	yellow	588	Disorders resulting from impaired renal function	green	766	Neuralgia, neuritis, and radiculitis NOS
turquoise	621	Endometrial hyperplasia	red	300.12	Agorophobia, social phobia, and panic disorder	brown	250.25	Diabetes type 2 with peripheral circulatory disorders	yellow	600	Hyperplasia of prostate	green	770	Myalgia and myositis unspecified
turquoise	622.1	Polyp of corpus uteri	red	300.3	Obsessive-compulsive disorders	brown	250.6	Polyneuropathy in diabetes	black	038	Septicemia	green	772	Symptoms of the muscles
turquoise	622.2	Mucous polyp of cervix	red	300.4	Dysthymic disorder	brown	250.7	Diabetic retinopathy	black	038.1	Gram negative septicemia			

Different modules are represented using different colours.

Modules can describe the interconnections among diseases, with those in the same module more tightly connected. In a further step of analysis, it is of interest to examine the interconnections among modules. The eigen-disease of each module is extracted for this purpose. Eigen-diseases are defined as the first principal components of the modules. Literature suggests that, under certain conditions, they have the highest connectivity and can best represent the corresponding modules. The hierarchical clustering of the nine eigen-diseases is shown in the right panel of Fig. S9 in SI. The brown and yellow eigen-diseases are clustered the first. Figure 4 suggests that this result is sensible as both modules are enriched with diseases related to endocrine and cardiovascular disorders. These two modules are then clustered with the black module, which is enriched with degenerative disorders. This connection has not been carefully examined for the Taiwan population in the literature and demands more attention.

### Temporal trends

As discussed above, a significant advantage of the eHDN is that temporal variations can be observed. For the studied diseases, their prevalence varies across time, as shown in the upper panel of Fig. 2. More importantly, their network structures, connectivity (bottom panel of Fig. 2), module structure (Figs 4 and S9), and hub structure (Table 1) all vary across time. For the module structure, which defines disease clustering/classification, we show detailed changes between consecutive years in Tables 3 and S4–S15 in SI. Take year 2012 and 2013 as an example (patterns for other years are similar). Most of the modules in 2012 have corresponding modules in 2013 with the Jaccard similarity indexes larger than 0.5 (which suggests a correspondence), expect for the red module in 2012, which has Jaccard index 0.36 with the magenta module in 2013. Overall it is observed that the module structures vary significantly between 2001 and 2005, become “stable” around 2005, and then fluctuate again between 2007 and 2011. Modules with “more unique” diseases, for example the modules enriched with gynaecological disorders (which have unique etiological pathways), tend to be more stable throughout the years. As an alternative way of visualising changes of module structures over time, the alluvial diagrams are shown in Figs S10–S22 in SI. It is noted that such plots provide similar information as in Tables S4–S15 in e SI, however, in a more intuitive way and can be preferred by some practitioners.

**Table 3:** Jaccard similarity index between modules in the year 2012 and 2013.

	module	2012									No. diseases
		grey	yellow	green	pink	blue	turquoise	black	brown	red	
2013	grey	0.94	0.00	0.00	0.00	0.00	0.01	0.00	0.01	0.02	1117
	black	0.00	0.64	0.00	0.00	0.00	0.01	0.00	0.00	0.00	24
	blue	0.00	0.00	0.83	0.00	0.00	0.00	0.00	0.00	0.00	32
	pink	0.00	0.00	0.00	1.00	0.00	0.00	0.00	0.00	0.00	22
	turquoise	0.00	0.00	0.00	0.00	0.91	0.00	0.00	0.00	0.00	39
	brown	0.00	0.02	0.00	0.00	0.00	0.50	0.00	0.00	0.00	27
	red	0.00	0.00	0.00	0.00	0.00	0.00	0.88	0.00	0.00	24
	green	0.00	0.00	0.00	0.00	0.00	0.00	0.00	0.61	0.00	25
	magenta	0.01	0.00	0.00	0.00	0.00	0.00	0.00	0.00	0.36	21
	yellow	0.00	0.15	0.00	0.00	0.00	0.26	0.00	0.00	0.00	25

**Figure 5:**
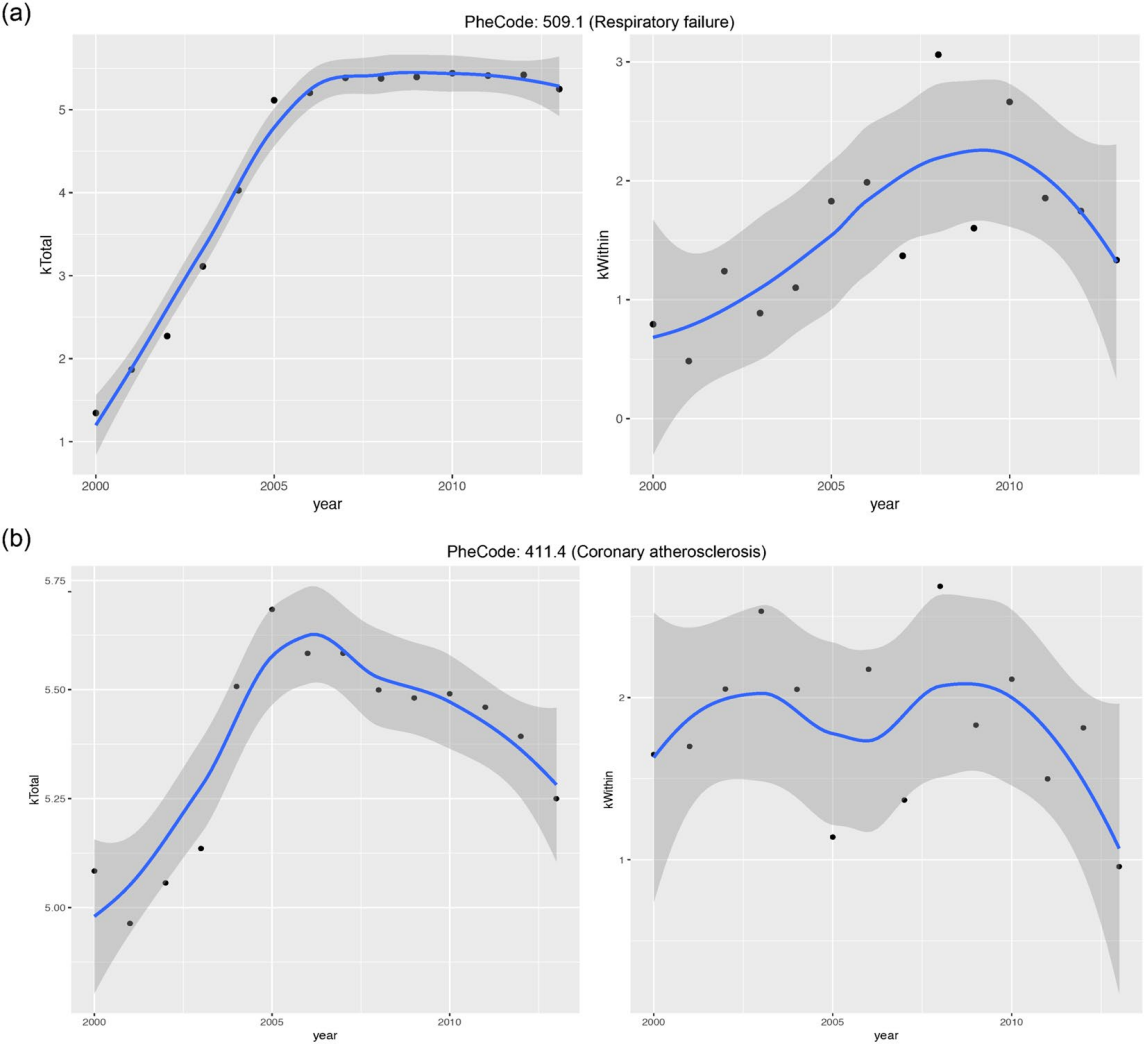
Connectivity changing patterns of respiratory failure (509.1) and coronary atherosclerosis (411.4) from 2000 to 2013.

For the modules, we also summarise their connectivity and examine changes over time. The results are presented in the radar charts in Figs S10–S22. Again, significant across-module differences are observed. For some modules, for example the one enriched with respiratory failure related diseases, significant temporal variations are observed. For disease 509.1 (respiratory failure), we present the temporal trends of connectivity and intramodular connectivity in the upper panel of Fig. 5. For a better visualisation, the nonparametric smooth fits are also added. The observed trends are similar to those reported in the literature^27^. A representative of “the opposite” is disease 411.4 (coronary atherosclerosis), which is shown in the bottom panel of Fig. 5 and has a much more stable connectivity. This observation is similar to that in Tseng *et al*.^26^.

## Discussion

HDN and other pan-disease research has drawn significant attention in recent literature and has brought significant insights beyond single-disease studies. Significantly different from the existing studies that are based on molecular information, in this study, we have taken advantage of the unique NHIRD, constructed the eHDN co-occurrence network, and studied its properties. This study has several contributions. The constructed eHDN provides a way of describing disease interconnections in a “global” manner. The adjacency measure establishes disease connections from an epidemiological perspective. The constructed modules provide an alternative way of disease clustering/classification. A closer examination of the analysis results suggest that the identified highly connected diseases and modules have sound biological interpretations, which provide support to the validity of the proposed analysis. This study also establishes a new way for analysing disease epidemiological data. The adopted technique is heavily based on the WCGNA studies. This study demonstrates the effectiveness of this technique for epidemiological data. In addition, this study also demonstrates various effective way of visualising the analysis results, which provides a more intuitive way of understanding disease epidemiological data. This study also provides an alternative analysis of NHIRD - in the literature, analysis has usually been focused on individual diseases.

Despite significant advancements, this study inevitably has limitations. The Taiwan population is dominatingly Asian. Thus, extending the findings to other races should be done with cautions. In our analysis, to describe the “big picture”, we conduct analysis on the whole selected cohort. The occurrence of most diseases depends on age, gender, and other factors. It will be of interest to conduct stratified analysis. Information is only available for the year 2000–2013. Without information on diagnosis prior to 2000, our analysis only captures disease occurrence within this time period. The WGCNA-based technique, although successful and popular, also has limitations. The network generated is undirected and hence cannot reflect the “order” of diseases. In this study, we have only analysed the most important network properties (connectivity, hub, module, etc.). Other, more subtle network properties may also be of interest. In addition, we have focused on the application of the WGCNA technique. Its theoretical validity for the NHIRD data has not been examined. However, the sensible analysis results provide some support to the validity of the analysis technique. There are other statistical techniques for network construction and analysis. It will be of interest, however beyond the scope of this study, to compare different network constructions for the NHIRD data.

The merit of HDN analysis has been well established in the literature. Results obtained in this study can be valuable for basic and clinical science researchers as well as health care providers and policymakers. This study focuses on disease connection from an epidemiological perspective and may well complement the existing HDN studies. Specifically, comparing the eHDN with molecular HDNs may suggest which disease connection are attributable to molecular and non-molecular causes. However, in the literature, there is a lack of molecular HDN specific to the Asian population (it is noted that molecular risk factors of many diseases are race-specific). In addition, the existing molecular HDNs have been constructed based on techniques other than the WGCNA. With these considerations, we postpone the joint analysis of eHDN and molecular HDNs to future studies.

## Electronic supplementary material

Supplementary Information
