# Supplementary material for: An Epidemiological Human Disease Network Derived from Disease Co-occurrence in Taiwan
Source: Sci Rep. 2018 Mar 14;8:4557. doi: 10.1038/s41598-018-21779-y (PMC5852024; doi:10.1038/s41598-018-21779-y)
Supplement: Supplementary file 1 — Supplementary Information [file 41598_2018_21779_MOESM1_ESM.pdf]

An Epidemiological Human Disease Network Derived from Disease Co-occurrence in Taiwan

Yefei Jiang[1], Shuangge Ma[2,†], Ben-Chang Shia[3], and Tian-Shyug Lee[1,*]

[1]Graduate Institute of Business Administration, College of Management, Fu Jen Catholic University, New Taipei City, 24205, Taiwan
[2]Yale School of Public Health, New Haven, Connecticut, United States of America
[3]College of Management, Taipei Medical University, Taipei, 11031, Taiwan
[*]036665@mail.fju.edu.tw
[†]shuangge.ma@yale.edu

Source Data and Study Population

The structure of EHR has been described in detail at the NHIRD website as well as in multiple publications[1, 2, 3]. Briefly, the EHR contains detailed information on patients' visits, including dates, medical care facilities and specialties, genders, dates of birth, and four major diagnoses coded in the International Classification of Disease, 9th Revision, Clinical Modification (ICD-9-CM) format[4, 5]. To protect privacy, the patient identities and institutions had been scrambled cryptographically. Each record contains information on the date of visit, a primary diagnosis, and two/four secondary diagnoses for outpatient/inpatient treatment, respectively.

As described in the main text, we obtained data on one million randomly selected subjects for the period of 2000-2013. This data selection has been determined with considerations on data quality and financial resource availability (access to NHIRD is not free, and the cost depends on the size of the required data).

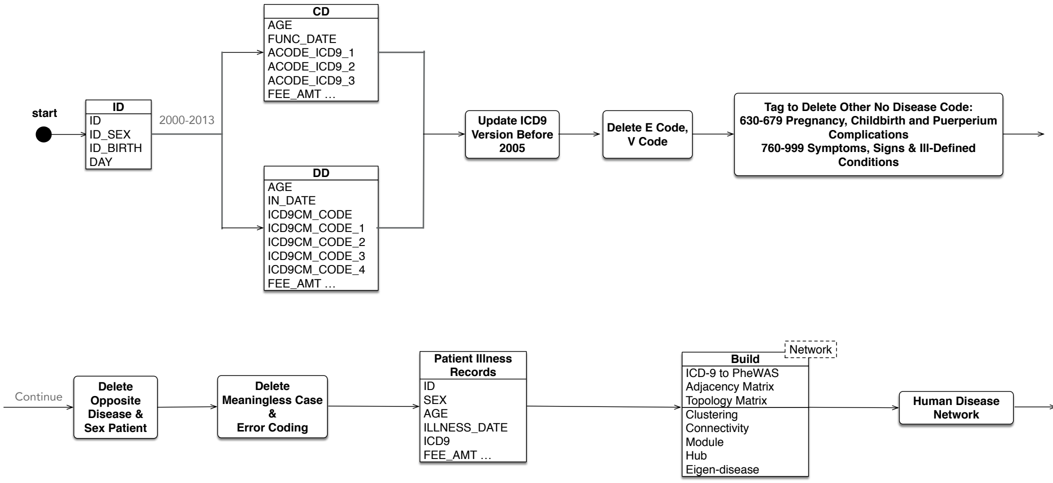

Figure S1: Flow chart of data processing.

Table S1: Number of patients by calendar year.

Year	Number of patients	Year	Number of patients
2000	736912	2007	806961
2001	756289	2008	796772
2002	778724	2009	800512
2003	785062	2010	797324
2004	818425	2011	802705
2005	834095	2012	795799
2006	812604	2013	790451

Table S2: Number and percentage of inpatient and outpatient treatments, by gender, age, and calendar year.

Variable		Inpatient		Outpatient	
		N	%	N	%
Gender					
	Female	632663	45.79	96364207	55.59
	Male	749086	54.21	76991518	44.41
Age					
	~19	155439	11.25	36968039	21.57
	20~29	90557	6.55	17599854	10.27
	30~39	141267	10.22	21496426	12.55
	40~49	192518	13.93	25016129	14.60
	50~59	214420	15.52	25675674	14.98
	60~69	197619	14.30	20771180	12.12
	70~	389929	28.22	23820220	13.90
Year					
	2000	64874	4.70	11640011	6.71
	2001	73217	5.30	11641557	6.72
	2002	80755	5.84	11629509	6.71
	2003	78076	5.65	11597504	6.69
	2004	96063	6.95	12894842	7.44
	2005	107636	7.79	13061504	7.53
	2006	105098	7.61	12321366	7.11
	2007	105602	7.64	12368636	7.13
	2008	107079	7.75	12253192	7.07
	2009	110516	8.00	12611191	7.27
	2010	112260	8.12	12593638	7.26
	2011	114298	8.27	12949864	7.47
	2012	113110	8.19	12912701	7.45
	2013	113165	8.19	12880210	7.43
		1381749	100.00	173355725	100.00

The flow chart of data processing is shown in Fig. S1 . It is noted that this processing is standard and comparable to those in the literature. In the first step, data were retrieved from both CD (for outpatient) and DD (for inpatient) files. The two files were merged using patient ID. As described in the main text, conversion of the 1992 ICD-9-CM version was conducted to achieve consistency. As the focus is on diseases, records corresponding to certain "non-disease" codes were removed from analysis. In the next step, certain inconsistent records were removed. The small number of such records suggests the high quality of data. Various ways of defining diseases based on the ICD-9-CM code have been discussed in the literature. The PheWAS Codes (PheCode) approach has been suggested in the literature as one of the "optimal" solutions[6, 7, 8, 9, 10, 11, 12]. In our data analysis, this approach can effectively reduce the number of diseases. For example, ICD-9-CM codes 250.00, 250.02, 250.20, 250.22, 250.30, 250.32, 250.80, 250.82, 250.90, and 250.92, which correspond to type-2 diabetes, are grouped into PheCode 250.2. With this conversion, the effective numbers of diseases from 2000 to 2013 are 1393, 1401, 1396, 1412, 1414, 1415, 1413, 1411, 1410, 1412, 1411, 1413, 1412, and 1410, respectively. As we are interested in comparing over time, a

disease is removed from analysis if it does not show up in all years. This processing leads to the removal of rare diseases, for which the counts are low and analysis may not be reliable. As described in the main text, the final number of diseases for analysis is 1,356. It is noted that not all subjects have treatments in all years. In Table S1, we present the numbers of patients that have treatments in specific years. In Table S2, we present the summary statistics on inpatient and outpatient treatments, stratified by gender, age, and calendar year. In Fig. S2, we show the heatmaps of the patient-disease matrix for two consecutive years. With a huge sample size, plots for all subjects have sizes too large. Thus, in Fig. S2, plotting is done for 1% of the subjects randomly selected from our dataset. Similar plots have been done for other years and are available from the authors. In Table S3, we provide the list of the top ten diseases with the highest prevalence.

Table S3: Top ten diseases with the highest prevalence.

PheCode	Disease	Prevalence (%)
465	Acute upper respiratory infections of multiple or unspecified sites	50.95
521.1	Dental caries	24.77
483	Acute bronchitis and bronchiolitis	21.24
464	Acute sinusitis	15.64
474.1	Acute tonsillitis	15.38
939	Atopic/contact dermatitis due to other or unspecified	13.41
523.3	Periodontitis (acute or chronic)	12.40
465.2	Acute pharyngitis	11.31
465.4	Acute laryngitis and tracheitis	10.84
523.1	Gingivitis	9.82

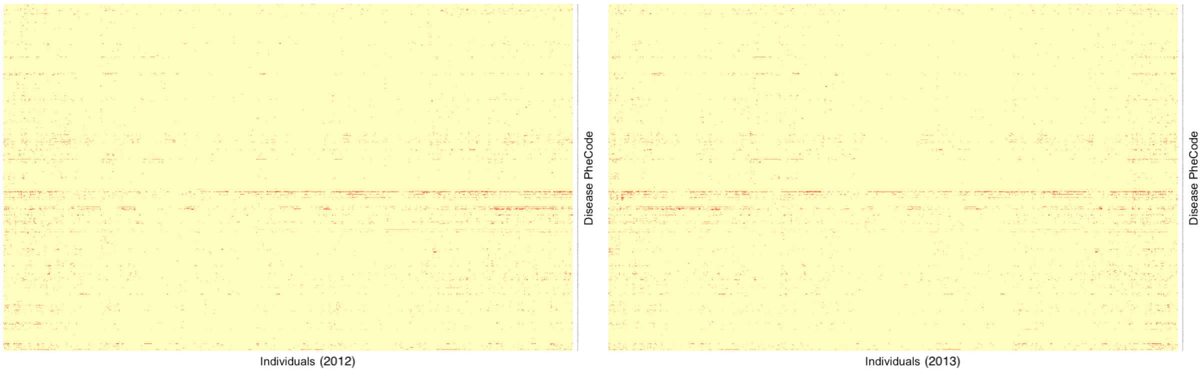

Figure S2: Heatmaps for the patient-disease matrix for 2012 and 2013. A red dot represents one disease occurrence.

Additional Analysis Results

Choosing the Thresholding Parameter τ

As described in the main text, τ is chosen sing the scale-free topology criterion, which has been extensively adopted in the literature[13, 14, 15, 16]. As shown in Fig. S3, we experimented with multiple threshold values and found that 0.03 led to a satisfactory scale-free topology fit with a high number of connections. For example, in Fig. S3, in 2013, the scale-free topology fit of TOM has $R^2 = 0.88$ and $\beta = -1.42$.

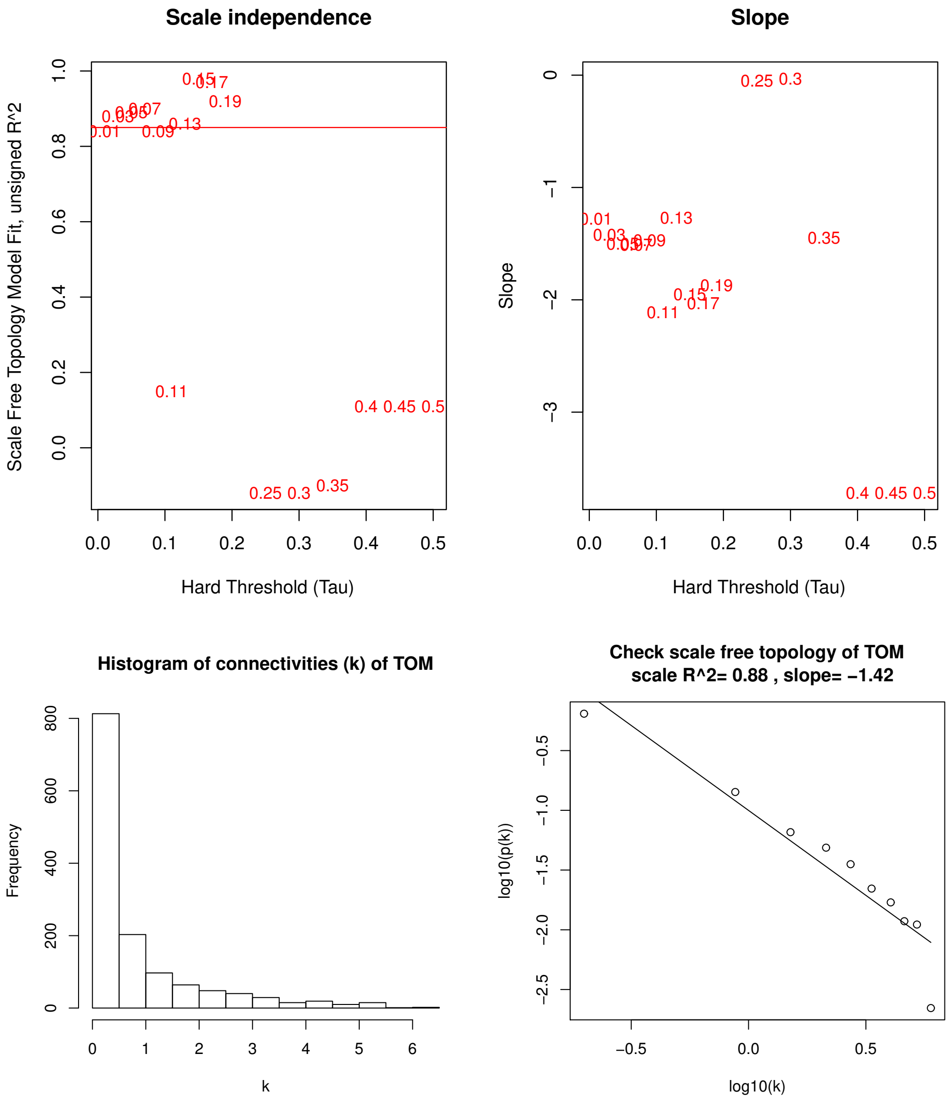

Figure S3: Scale-free topology check (year 2013): scale-free topology fitting plot with R^2 for different threshold values (top left), slope (top right), histogram of connectivity values (bottom left), and scale-free topology fitting with R^2.

Correlation Between the Two Connectivity Measures

As described in the main text, there are two common definitions of connectivity. In Fig. S4, it is shown that for the analysed data, the two connectivity measures are highly correlated. In our analysis, we adopted weighted connectivity $k_i = \sum_{j \neq i} TOM_{ij}$.

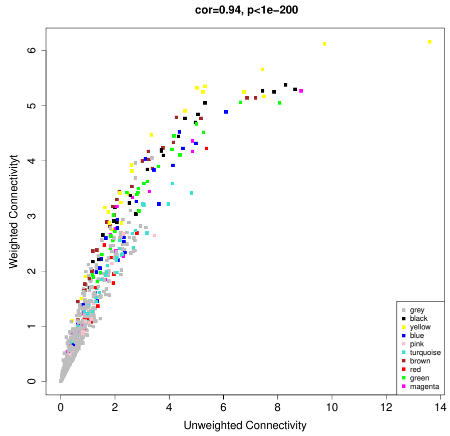

Figure S4: Scatter plot of weighted connectivity and unweighted connectivity (year 2013).

Network Construction and Module Detection

In Fig. S5, we present the summary of TOM connectivity, for both connectivity and intramodular connectivity, and for different modules. The differences across modules are clearly observed.

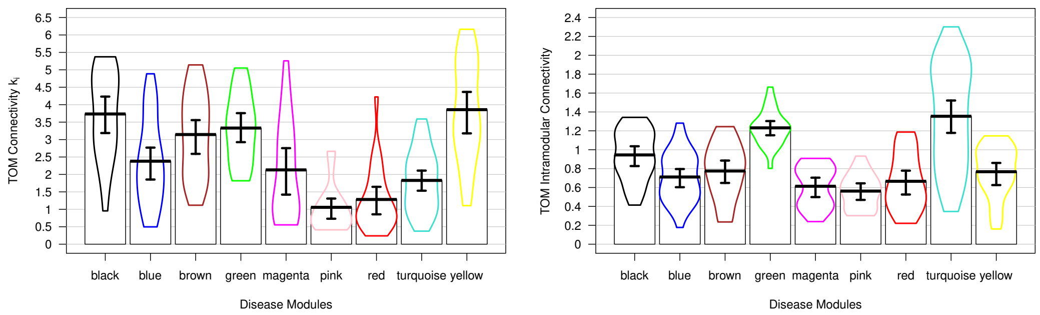

Figure S5: Scatter plot of weighted connectivity and unweighted connectivity (year 2013).

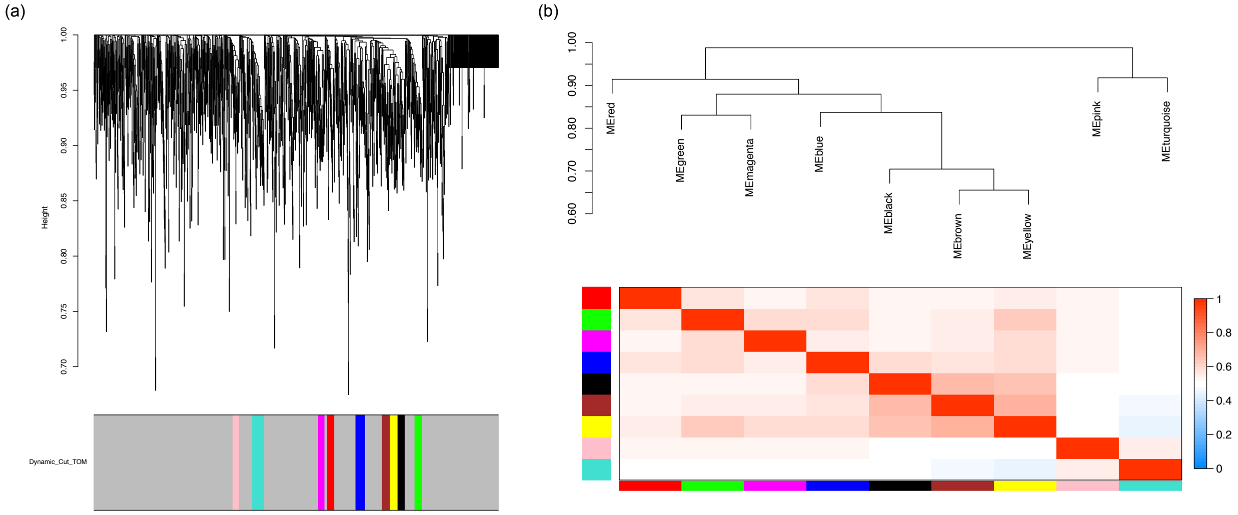

Figure S6: Dendrograms and heatmaps of diseases (a) and module eigen-diseases (b), for year 2013. Different colours correspond to different modules. Grey represents diseases not belonging to any identified module.

In Fig. S7, node size represents the TOM connectivity of each diseases, with a larger node means higher connectivity value. Edges is the connection between diseases. Disease nodes coloured by module colour from hierarchical clustering. Hence, the nodes within the same disease module share same the colour. Also, noted is that many of the co-occurrences we found were already well known; the selection of modules is noteworthy for exploration that also requires a clinical medicine background.

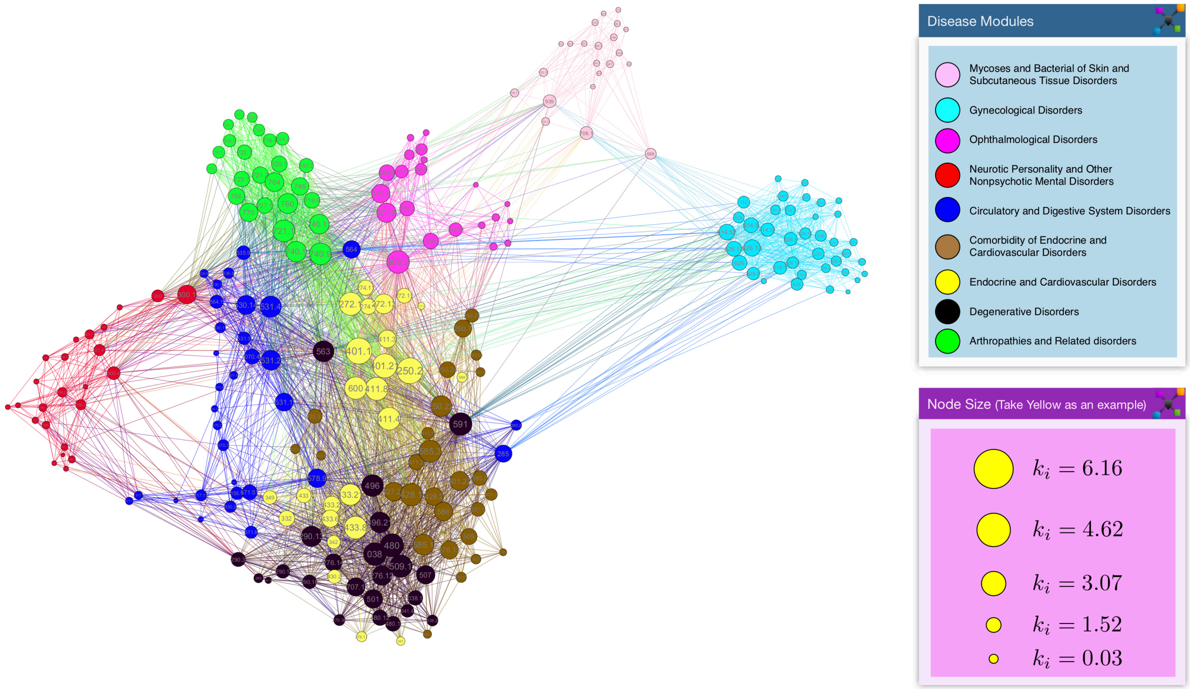

Figure S7: Module disease network in the year 2013.

In Fig. S8, we present the scatter plot of the module membership versus intramodular connectivity. The correlation coefficients are highly significant for all modules, suggesting that disease share almost the same comorbidity as their neighbours.

.

Table S4: Jaccard similarity index between modules in year 2000 and 2001.

		2000											No. diseases
	module	grey	black	turquoise	blue	purple	magenta	red	brown	green	pink	yellow	
2001	grey	0.89	0.00	0.01	0.00	0.02	0.02	0.00	0.01	0.00	0.02	0.00	1131
	yellow	0.01	0.40	0.00	0.00	0.00	0.00	0.00	0.00	0.00	0.00	0.00	26
	turquoise	0.01	0.10	0.47	0.00	0.00	0.00	0.01	0.00	0.01	0.00	0.00	51
	brown	0.00	0.00	0.00	0.95	0.00	0.00	0.00	0.00	0.00	0.00	0.00	39
	green	0.00	0.00	0.00	0.00	0.00	0.00	0.74	0.00	0.00	0.00	0.00	24
	black	0.00	0.00	0.00	0.00	0.00	0.00	0.00	0.72	0.00	0.00	0.00	22
	red	0.00	0.00	0.01	0.00	0.00	0.00	0.00	0.00	0.74	0.00	0.00	23
	blue	0.01	0.00	0.13	0.00	0.00	0.00	0.00	0.00	0.00	0.00	0.51	40

Table S5: Jaccard similarity index between modules in year 2001 and 2002.

		2001								No. diseases
	module	yellow	grey	turquoise	brown	blue	green	black	red	
2002	brown	0.93	0.00	0.00	0.00	0.00	0.00	0.00	0.00	26
	grey	0.00	0.89	0.02	0.01	0.00	0.00	0.01	0.01	1132
	turquoise	0.00	0.00	0.39	0.00	0.02	0.00	0.00	0.24	49
	yellow	0.00	0.00	0.00	0.62	0.00	0.00	0.00	0.00	24
	blue	0.00	0.01	0.00	0.00	0.71	0.00	0.00	0.00	44
	green	0.00	0.00	0.01	0.00	0.00	0.73	0.00	0.00	21
	pink	0.00	0.01	0.00	0.00	0.00	0.00	0.31	0.00	20
	black	0.00	0.02	0.00	0.00	0.00	0.00	0.00	0.00	20
	red	0.00	0.02	0.00	0.00	0.00	0.00	0.00	0.00	20

Table S6: Jaccard similarity index between modules in year 2002 and 2003.

		2002									No. diseases
	module	brown	grey	turquoise	red	yellow	green	black	blue	pink	
2003	green	0.96	0.00	0.00	0.00	0.00	0.00	0.00	0.00	0.00	25
	grey	0.00	0.92	0.00	0.00	0.00	0.00	0.00	0.00	0.01	1083
	turquoise	0.00	0.02	0.59	0.00	0.00	0.03	0.00	0.00	0.00	72
	pink	0.00	0.00	0.00	0.82	0.00	0.00	0.00	0.00	0.00	20
	blue	0.00	0.01	0.00	0.00	0.73	0.00	0.00	0.00	0.00	33
	brown	0.00	0.01	0.00	0.00	0.00	0.50	0.00	0.00	0.00	30
	black	0.00	0.00	0.00	0.00	0.00	0.00	0.76	0.00	0.00	24
	yellow	0.00	0.00	0.00	0.00	0.00	0.00	0.00	0.57	0.00	25
	magenta	0.00	0.00	0.00	0.00	0.00	0.00	0.00	0.31	0.00	20
	red	0.00	0.01	0.00	0.00	0.00	0.00	0.00	0.00	0.33	24

Table S7: Jaccard similarity index between modules in year 2003 and 2004.

		2003										No. diseases
	module	green	grey	turquoise	brown	pink	blue	black	yellow	magenta	red	
2004	red	0.82	0.00	0.00	0.00	0.00	0.00	0.00	0.00	0.00	0.00	26
	grey	0.00	0.89	0.01	0.00	0.02	0.00	0.00	0.00	0.01	0.01	1073
	turquoise	0.01	0.00	0.61	0.00	0.00	0.00	0.00	0.00	0.00	0.00	49
	yellow	0.00	0.00	0.03	0.69	0.02	0.00	0.00	0.00	0.00	0.00	31
	brown	0.00	0.00	0.00	0.00	0.00	0.86	0.00	0.00	0.00	0.00	36
	magenta	0.00	0.00	0.00	0.00	0.00	0.00	0.83	0.00	0.00	0.00	20
	blue	0.00	0.01	0.00	0.00	0.00	0.00	0.00	0.54	0.22	0.00	46
	pink	0.00	0.02	0.01	0.00	0.00	0.00	0.00	0.00	0.00	0.00	24
	black	0.00	0.01	0.07	0.00	0.00	0.00	0.07	0.00	0.00	0.00	24
	green	0.00	0.01	0.00	0.00	0.00	0.00	0.00	0.00	0.00	0.50	27

Table S8: Jaccard similarity index between modules in year 2004 and 2005.

		2004										
	module	red	grey	turquoise	yellow	pink	brown	magenta	black	blue	green	No. diseases
2005	black	0.79	0.00	0.01	0.00	0.00	0.00	0.00	0.00	0.00	0.00	24
	grey	0.00	0.91	0.02	0.00	0.00	0.00	0.00	0.01	0.01	0.00	1061
	purple	0.00	0.00	0.41	0.00	0.00	0.00	0.00	0.00	0.00	0.00	20
	blue	0.01	0.00	0.10	0.68	0.00	0.00	0.00	0.00	0.00	0.00	43
	red	0.00	0.00	0.00	0.00	0.92	0.00	0.00	0.00	0.00	0.00	24
	brown	0.00	0.00	0.00	0.00	0.00	0.95	0.00	0.00	0.00	0.00	38
	yellow	0.00	0.00	0.00	0.00	0.00	0.00	0.80	0.09	0.00	0.00	25
	turquoise	0.00	0.00	0.00	0.00	0.00	0.00	0.00	0.16	0.67	0.00	54
	green	0.00	0.00	0.00	0.00	0.00	0.00	0.00	0.00	0.00	0.73	25
	magenta	0.00	0.02	0.00	0.00	0.00	0.00	0.00	0.00	0.00	0.00	21
	pink	0.00	0.02	0.00	0.00	0.00	0.00	0.00	0.00	0.00	0.00	21

Table S9: Jaccard similarity index between modules in year 2005 and 2006.

		2005											
	module	black	grey	blue	red	pink	brown	magenta	purple	yellow	turquoise	green	No. diseases
2006	black	0.88	0.00	0.00	0.00	0.00	0.00	0.00	0.00	0.00	0.00	0.00	23
	grey	0.00	0.89	0.00	0.00	0.02	0.00	0.02	0.00	0.00	0.00	0.02	1099
	yellow	0.00	0.00	0.60	0.00	0.00	0.00	0.00	0.00	0.00	0.00	0.00	29
	green	0.00	0.00	0.00	0.86	0.00	0.00	0.00	0.00	0.00	0.00	0.00	28
	brown	0.00	0.01	0.00	0.00	0.00	0.82	0.00	0.00	0.00	0.00	0.00	44
	turquoise	0.01	0.02	0.13	0.00	0.00	0.00	0.00	0.36	0.00	0.00	0.00	55
	red	0.00	0.00	0.00	0.00	0.00	0.00	0.00	0.00	0.89	0.00	0.00	26
	blue	0.00	0.00	0.00	0.00	0.00	0.00	0.00	0.00	0.00	0.86	0.00	5

Table S10: Jaccard similarity index between modules in year 2006 and 2007.

		2006								
	module	black	grey	yellow	turquoise	green	brown	red	blue	No. diseases
2007	black	0.92	0.00	0.00	0.00	0.00	0.00	0.00	0.00	25
	grey	0.00	0.87	0.00	0.02	0.01	0.02	0.00	0.00	1051
	yellow	0.00	0.00	0.30	0.19	0.00	0.00	0.00	0.00	27
	blue	0.00	0.00	0.22	0.29	0.00	0.00	0.00	0.00	33
	red	0.00	0.00	0.00	0.00	0.71	0.00	0.00	0.00	25
	green	0.00	0.00	0.00	0.00	0.00	0.59	0.00	0.00	26
	pink	0.00	0.00	0.00	0.00	0.00	0.00	0.92	0.00	24
	turquoise	0.00	0.00	0.00	0.00	0.00	0.00	0.00	0.88	55
	greenyellow	0.00	0.02	0.00	0.00	0.00	0.00	0.00	0.00	20
	purple	0.00	0.02	0.00	0.00	0.00	0.00	0.00	0.00	20
	brown	0.00	0.03	0.00	0.00	0.00	0.00	0.00	0.00	29
	magenta	0.00	0.02	0.06	0.00	0.00	0.00	0.00	0.00	21

Table S11: Jaccard similarity index between modules in year 2007 and 2008.

		2007												
	module	black	grey	yellow	red	greenyellow	green	blue	purple	pink	turquoise	brown	magenta	No. diseases
2008	grey	0.02	0.83	0.00	0.00	0.00	0.00	0.00	0.02	0.00	0.02	0.01	0.02	1063
	turquoise	0.00	0.02	0.27	0.00	0.00	0.00	0.43	0.00	0.00	0.00	0.00	0.00	77
	yellow	0.00	0.01	0.00	0.63	0.00	0.00	0.00	0.00	0.00	0.00	0.00	0.00	32
	black	0.00	0.00	0.00	0.00	0.83	0.00	0.00	0.00	0.00	0.00	0.00	0.00	24
	blue	0.00	0.02	0.00	0.00	0.00	0.57	0.00	0.00	0.00	0.00	0.00	0.00	46
	red	0.00	0.00	0.00	0.00	0.00	0.00	0.00	0.00	0.96	0.00	0.00	0.00	25
	brown	0.00	0.00	0.00	0.00	0.00	0.00	0.00	0.00	0.00	0.64	0.00	0.00	40
	green	0.00	0.01	0.00	0.00	0.00	0.00	0.00	0.00	0.00	0.00	0.49	0.00	26
	pink	0.00	0.02	0.00	0.00	0.00	0.00	0.00	0.00	0.00	0.00	0.00	0.00	23

Table S12: Jaccard similarity index between modules in year 2008 and 2009.

		2008									
	module	grey	turquoise	yellow	black	blue	pink	red	brown	green	No. diseases
2009	grey	0.87	0.00	0.03	0.00	0.01	0.02	0.00	0.00	0.01	1090
	turquoise	0.00	0.50	0.00	0.00	0.00	0.00	0.00	0.00	0.00	43
	yellow	0.00	0.41	0.00	0.00	0.00	0.00	0.00	0.00	0.00	33
	red	0.00	0.00	0.00	0.88	0.00	0.00	0.00	0.00	0.00	23
	brown	0.00	0.00	0.00	0.00	0.83	0.00	0.00	0.00	0.00	40
	pink	0.00	0.00	0.00	0.00	0.00	0.00	0.84	0.00	0.00	21
	blue	0.00	0.00	0.00	0.00	0.00	0.00	0.00	0.95	0.00	40
	green	0.01	0.00	0.00	0.00	0.00	0.00	0.00	0.00	0.32	24
	black	0.02	0.00	0.00	0.00	0.00	0.00	0.00	0.00	0.00	22
	magenta	0.02	0.00	0.00	0.00	0.00	0.00	0.00	0.00	0.00	20

Table S13: Jaccard similarity index between modules in year 2009 and 2010.

		2009										
	module	black	grey	yellow	turquoise	red	brown	pink	blue	green	magenta	No. diseases
2010	black	0.91	0.00	0.00	0.00	0.00	0.00	0.00	0.00	0.00	0.00	22
	grey	0.00	0.90	0.00	0.00	0.00	0.00	0.00	0.00	0.01	0.02	1047
	turquoise	0.00	0.01	0.41	0.27	0.00	0.00	0.00	0.01	0.00	0.03	71
	purple	0.00	0.00	0.00	0.42	0.00	0.00	0.00	0.00	0.02	0.00	21
	red	0.00	0.00	0.00	0.00	0.85	0.00	0.00	0.00	0.00	0.00	25
	green	0.00	0.00	0.00	0.00	0.00	0.63	0.00	0.00	0.00	0.00	25
	pink	0.00	0.00	0.00	0.00	0.00	0.00	0.95	0.00	0.00	0.00	22
	blue	0.00	0.00	0.00	0.00	0.00	0.00	0.00	0.95	0.00	0.00	40
	yellow	0.00	0.01	0.00	0.00	0.00	0.00	0.00	0.00	0.34	0.00	27
	magenta	0.00	0.01	0.00	0.00	0.00	0.33	0.00	0.00	0.00	0.00	21
	brown	0.00	0.03	0.00	0.00	0.00	0.00	0.00	0.00	0.00	0.00	35

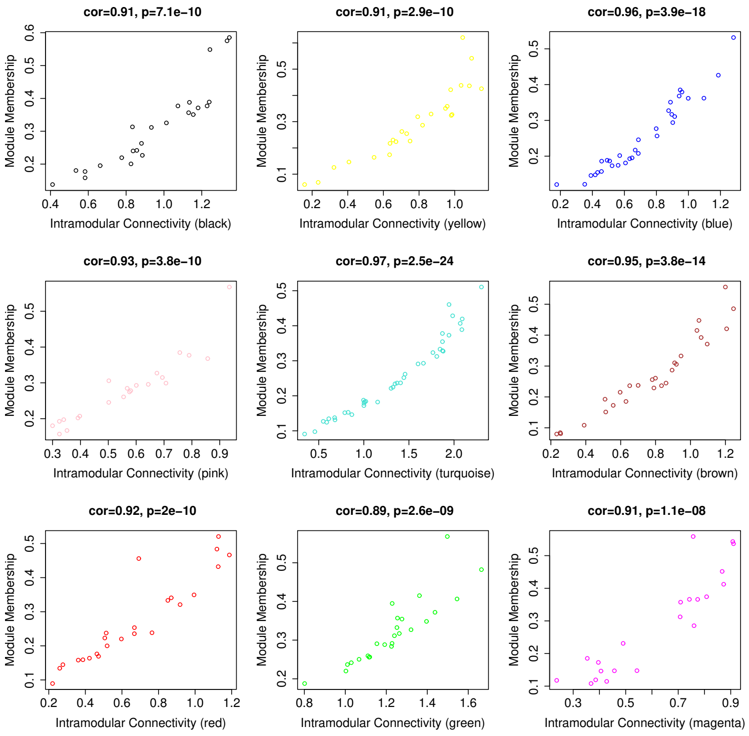

Figure S8: Module membership (k^q_{ME}) versus intramodular connectivity (k^q) in each disease modules (2013).

References

[1] Chen, P. *et al.* Carbamazepine-induced toxic effects and hla-b* 1502 screening in taiwan. *New England Journal of Medicine* **364**, 1126–1133 (2011).

[2] Wu, C.-Y. *et al.* Association between nucleoside analogues and risk of hepatitis b virus–related hepatocellular carcinoma recurrence following liver resection. *Jama* **308**, 1906–1913 (2012).

[3] Chen, W. *et al.* Incidence and outcomes of acute respiratory distress syndrome: a nationwide registry-based study in taiwan, 1997 to 2011. *Medicine* **94** (2015).

[4] National Health Research Institutes. National health insurance research database (NHIRD). `http://nhird.nhri.org.tw/`. Online; accessed 19 Aprial 2017.

Table S14: Jaccard similarity index between modules in year 2010 and 2011.

		2010											
	module	black	grey	turquoise	brown	red	green	magenta	purple	pink	blue	yellow	No. diseases
	red	0.76	0.00	0.05	0.00	0.00	0.00	0.00	0.00	0.00	0.00	0.00	29
	grey	0.00	0.91	0.01	0.03	0.00	0.00	0.00	0.00	0.00	0.00	0.01	1082
	brown	0.00	0.00	0.42	0.00	0.00	0.00	0.00	0.13	0.00	0.00	0.00	41
	pink	0.00	0.00	0.00	0.00	0.84	0.00	0.00	0.00	0.00	0.00	0.00	21
2011	blue	0.00	0.00	0.00	0.00	0.00	0.48	0.43	0.00	0.00	0.00	0.00	46
	yellow	0.00	0.00	0.28	0.00	0.00	0.00	0.00	0.23	0.00	0.00	0.00	33
	black	0.00	0.00	0.00	0.00	0.00	0.00	0.00	0.00	0.91	0.00	0.00	22
	turquoise	0.00	0.01	0.00	0.00	0.00	0.00	0.00	0.00	0.00	0.69	0.00	53
	green	0.00	0.01	0.00	0.00	0.00	0.00	0.00	0.02	0.00	0.00	0.56	29

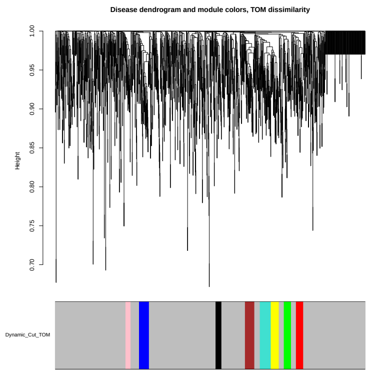

(a) Clustering dendrogram of TOM dissimilarity.

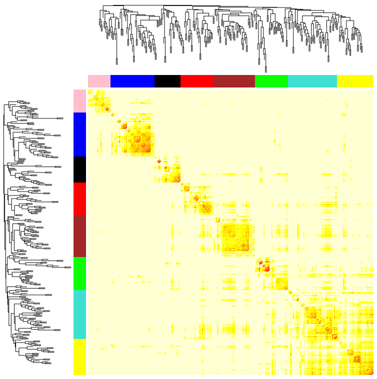

(b) Heatmap of module diseases.

Figure S9: Disease module dendrogram (left) and heatmap (right), for year 2012.

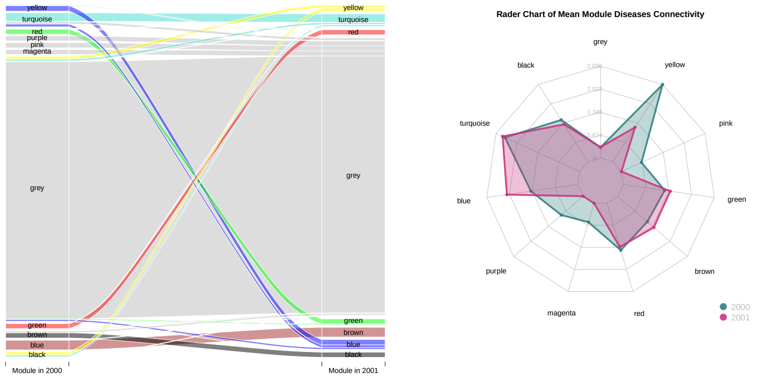

Figure S10: Changing patterns of disease modules between year 2000 and 2001. Left: alluvial diagram. Blocks represent modules, and stream lines between blocks represent changes in the composition of modules. Right: Radar chart of the mean connectivity of each module.

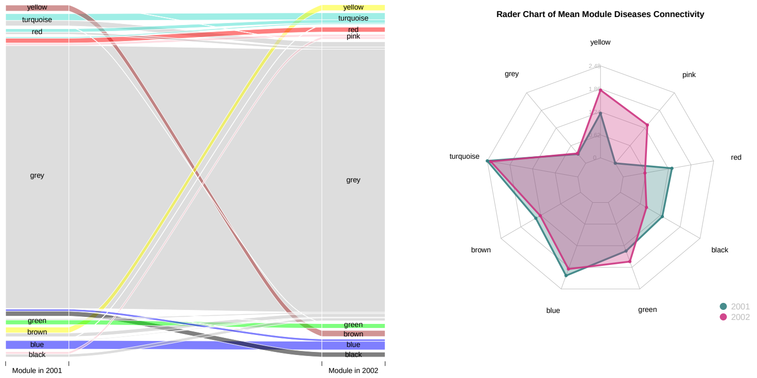

Figure S11: Changing patterns of disease modules between year 2001 and 2002. Left: alluvial diagram. Blocks represent modules, and stream lines between blocks represent changes in the composition of modules. Right: Radar chart of the mean connectivity of each module.

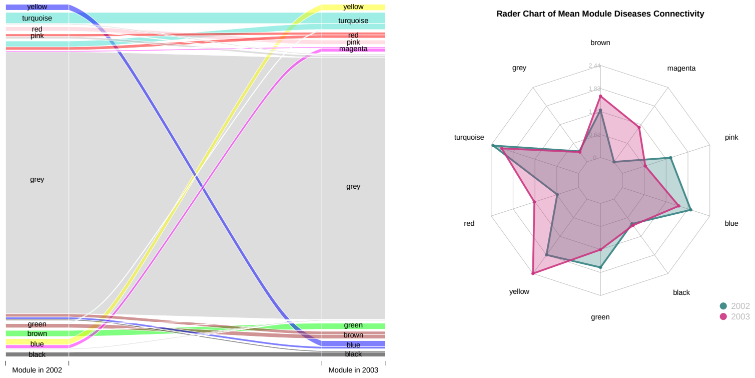

Figure S12: Changing patterns of disease modules between year 2002 and 2003. Left: alluvial diagram. Blocks represent modules, and stream lines between blocks represent changes in the composition of modules. Right: Radar chart of the mean connectivity of each module.

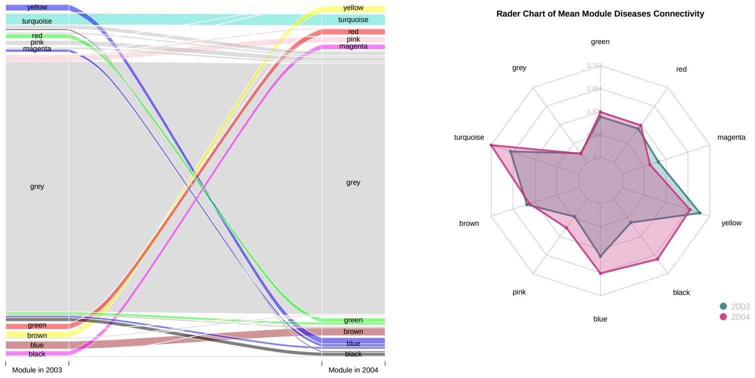

Figure S13: Changing patterns of disease modules between year 2003 and 2004. Left: alluvial diagram. Blocks represent modules, and stream lines between blocks represent changes in the composition of modules. Right: Radar chart of the mean connectivity of each module.

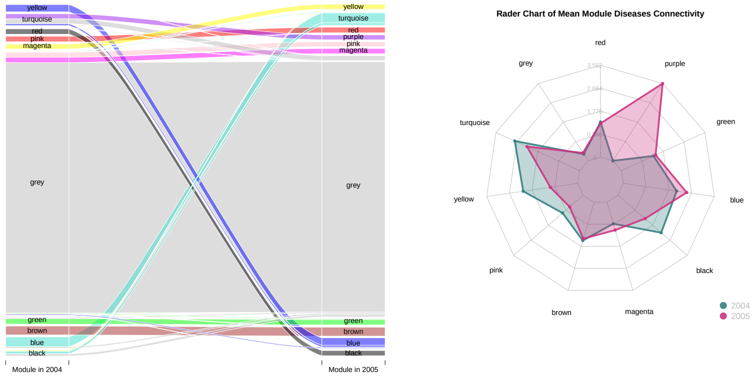

Figure S14: Changing patterns of disease modules between year 2004 and 2005. Left: alluvial diagram. Blocks represent modules, and stream lines between blocks represent changes in the composition of modules. Right: Radar chart of the mean connectivity of each module.

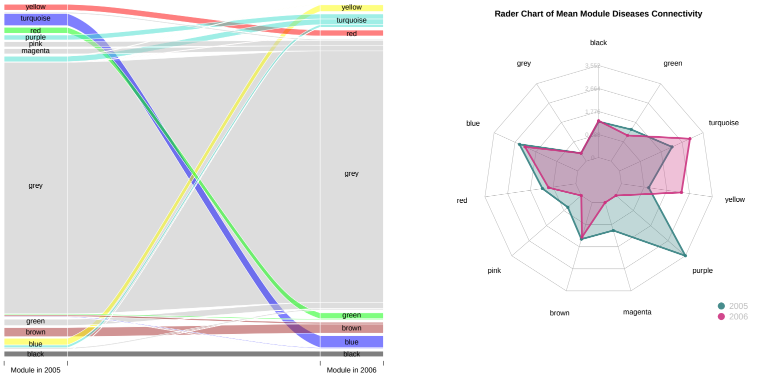

Figure S15: Changing patterns of disease modules between year 2005 and 2006. Left: alluvial diagram. Blocks represent modules, and stream lines between blocks represent changes in the composition of modules. Right: Radar chart of the mean connectivity of each module.

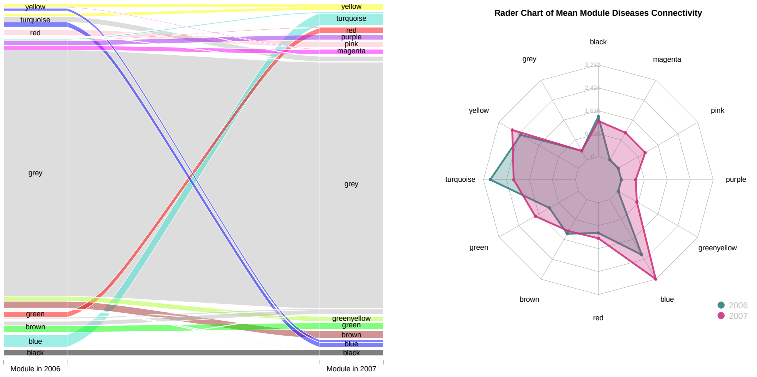

Figure S16: Changing patterns of disease modules between year 2006 and 2007. Left: alluvial diagram. Blocks represent modules, and stream lines between blocks represent changes in the composition of modules. Right: Radar chart of the mean connectivity of each module.

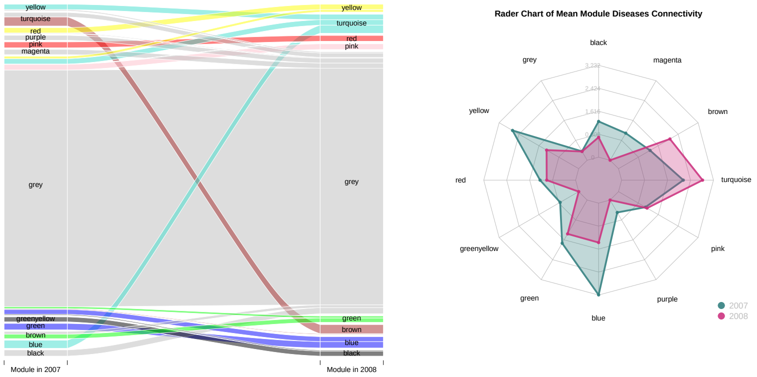

Figure S17: Changing patterns of disease modules between year 2007 and 2008. Left: alluvial diagram. Blocks represent modules, and stream lines between blocks represent changes in the composition of modules. Right: Radar chart of the mean connectivity of each module.

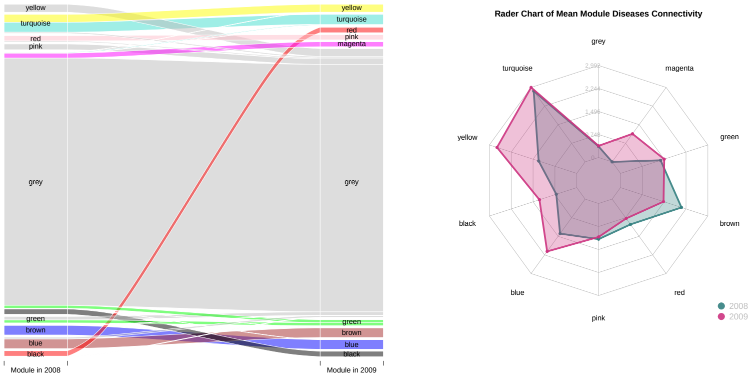

Figure S18: Changing patterns of disease modules between year 2008 and 2009. Left: alluvial diagram. Blocks represent modules, and stream lines between blocks represent changes in the composition of modules. Right: Radar chart of the mean connectivity of each module.

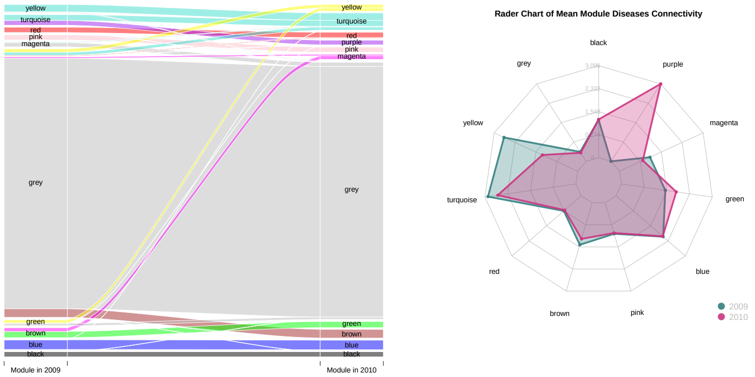

Figure S19: Changing patterns of disease modules between year 2009 and 2010. Left: alluvial diagram. Blocks represent modules, and stream lines between blocks represent changes in the composition of modules. Right: Radar chart of the mean connectivity of each module.

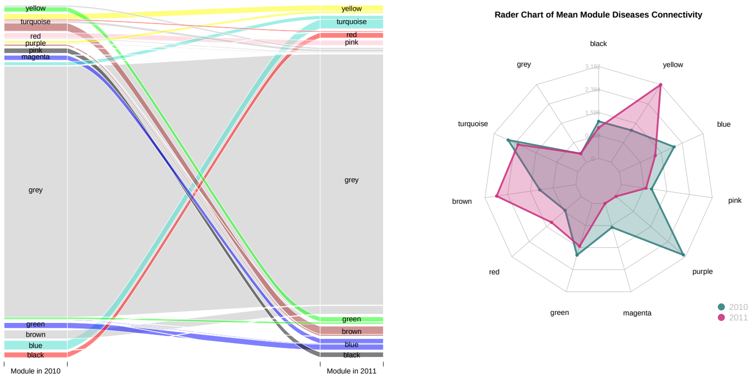

Figure S20: Changing patterns of disease modules between year 2010 and 2011. Left: alluvial diagram. Blocks represent modules, and stream lines between blocks represent changes in the composition of modules. Right: Radar chart of the mean connectivity of each module.

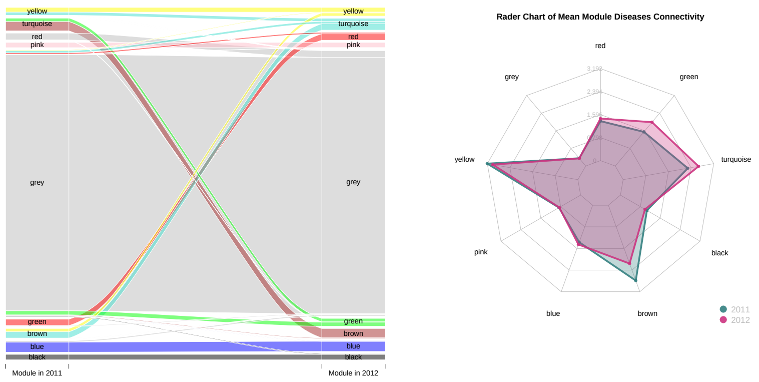

Figure S21: Changing patterns of disease modules between year 2011 and 2012. Left: alluvial diagram. Blocks represent modules, and stream lines between blocks represent changes in the composition of modules. Right: Radar chart of the mean connectivity of each module.

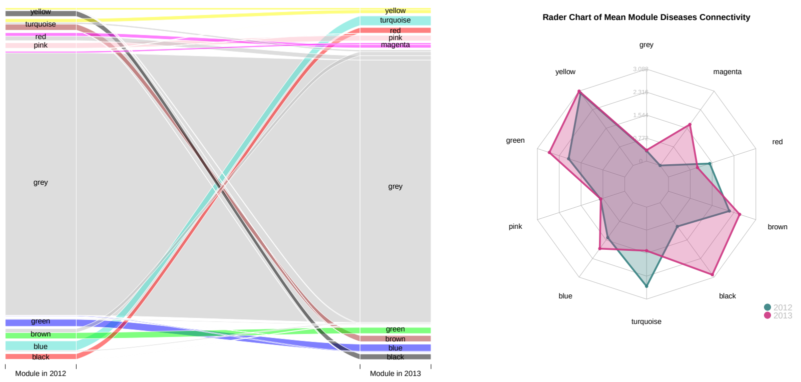

Figure S22: Changing patterns of disease modules between year 2012 and 2013. Left: alluvial diagram. Blocks represent modules, and stream lines between blocks represent changes in the composition of modules. Right: Radar chart of the mean connectivity of each module.

Table S15: Jaccard similarity index between modules in year 2011 and 2012.

		2011									No. diseases
	module	red	grey	yellow	pink	blue	brown	black	turquoise	green	
	grey	0.03	0.93	0.00	0.00	0.00	0.00	0.00	0.00	0.00	1078
	yellow	0.00	0.00	0.39	0.00	0.00	0.21	0.00	0.00	0.00	35
	pink	0.00	0.00	0.00	0.95	0.00	0.00	0.00	0.00	0.00	22
	blue	0.00	0.00	0.00	0.00	0.85	0.00	0.00	0.00	0.00	43
2012	turquoise	0.00	0.01	0.19	0.00	0.00	0.41	0.00	0.00	0.00	48
	black	0.00	0.00	0.00	0.00	0.00	0.00	0.88	0.00	0.00	25
	brown	0.00	0.00	0.00	0.00	0.00	0.00	0.00	0.68	0.00	41
	red	0.00	0.01	0.00	0.00	0.00	0.00	0.00	0.00	0.74	32
	green	0.02	0.02	0.00	0.00	0.00	0.00	0.00	0.20	0.00	32

[5] Centers for Disease Control and Prevention and others. International classification of diseases, ninth revision, clinical modification (icd-9-cm). *URL: http://www. cdc. gov/nchs/about/otheract/icd9/abticd9. htm [accessed 2004 Dec 16]* (2013).

[6] Denny, J. C. *et al.* Phewas: demonstrating the feasibility of a phenome-wide scan to discover gene–disease associations. *Bioinformatics* **26**, 1205–1210 (2010).

[7] Warner, J. L. & Alterovitz, G. Phenome-based analysis as a means for discovering context-dependent clinical reference ranges. In *AMIA* (2012).

[8] Denny, J. C. *et al.* Systematic comparison of phenome-wide association study of electronic medical record data and genome-wide association study data. *Nature biotechnology* **31**, 1102–1111 (2013).

[9] Hebbring, S. J. The challenges, advantages and future of phenome-wide association studies. *Immunology* **141**, 157–165 (2014).

[10] Rastegar-Mojarad, M., Ye, Z., Kolesar, J. M., Hebbring, S. J. & Lin, S. M. Opportunities for drug repositioning from phenome-wide association studies. *Nature biotechnology* **33**, 342–345 (2015).

[11] Chen, Y. *et al.* Building bridges across electronic health record systems through inferred phenotypic topics. *Journal of biomedical informatics* **55**, 82–93 (2015).

[12] Prieto, M. *et al.* Leveraging electronic health records to study pleiotropic effects on bipolar disorder and medical comorbidities. *Translational Psychiatry* **6**, e870 (2016).

[13] Zhang, B., Horvath, S. *et al.* A general framework for weighted gene co-expression network analysis. *Statistical applications in genetics and molecular biology* **4**, 1128 (2005).

[14] Dong, J. & Horvath, S. Understanding network concepts in modules. *BMC systems biology* **1**, 24 (2007).

[15] Goh, K.-I. *et al.* The human disease network. *Proceedings of the National Academy of Sciences* **104**, 8685–8690 (2007).

[16] Hidalgo, C. A., Blumm, N., Barabási, A.-L. & Christakis, N. A. A dynamic network approach for the study of human phenotypes. *PLoS computational biology* **5**, e1000353 (2009).
